# Childhood Centeredness is a Broader Predictor of Young Adulthood Mental Health than Childhood Adversity, Attachment, and Other Positive Childhood Experiences

**DOI:** 10.1007/s42844-023-00089-x

**Published:** 2023-03-23

**Authors:** Angela J. Narayan, Donald E. Frederick, Jillian S. Merrick, Madison D. Sayyah, Matthew D. Larson

**Affiliations:** 1 Department of Psychology, University of Denver, Denver, USA; 2 Institute for Quantitative Social Science, Harvard University, Cambridge, USA; 3 The Human Improvement Project, Boulder, USA

**Keywords:** Centeredness, ACEs, BCEs, Attachment, Young adulthood, Diversity, Inclusivity

## Abstract

This study introduced the novel concept of Centeredness, a measure of the emotional atmosphere of the family of origin and a target adult individual’s perception of feeling safe, accepted, and supported from childhood primary caregivers and other family members. This study developed a Centeredness scale for adult respondents and tested hypotheses that higher levels of overall Centeredness would predict lower levels of depression and anxiety symptoms; suicidal thoughts and behaviors (STBs); and aggressive behavior; and higher levels of life satisfaction. Predictive effects of Centeredness were compared against attachment-related anxiety and avoidance, and adverse and benevolent childhood experiences (ACEs and BCEs). Participants were recruited via the Prolific-Academic (Pro-A) survey panel into two large independent samples of US young adults aged 19–35 years [Sample 1 (test sample), *N* = 548, 53.5% female, 2.2% gender non-conforming, 68.3% White, recruited before the pandemic; Sample 2 (replication sample), *N* = 1,198, 56.2% female, 2.3% gender non-conforming, 66.4% White; recruited during the pandemic]. Participants completed the novel Centeredness scale, which showed strong psychometric properties, and standardized, publicly available assessments of childhood experiences and mental health outcomes. Centeredness was the only variable that significantly predicted each mental health outcome across both samples. BCEs predicted all outcomes except aggressive behavior in the test sample. Centeredness and BCEs were also the only two variables that significantly predicted a dimensional mental health composite in both samples. Neither attachment-related anxiety and avoidance nor ACEs were as broadly predictive. The Centeredness scale assesses emotional aspects of childhood family relationships with individuals of diverse backgrounds and family compositions. Clinical and cultural implications are discussed.

**Supplementary Information:**

The online version contains supplementary material available at 10.1007/s42844-023-00089-x.

## Introduction

The perceptions of having a positive, safe, and supportive home atmosphere within one’s family are historically well-documented to be universally helpful qualities for all developing children to thrive. Indeed, well-replicated research across developmental, clinical, social, and family psychology converges on findings that children who grow up feeling connected to and loved and accepted by their families and perceive a strong sense of belonging are more likely to show multi-dimensional competence (Masten, 2001; Thompson, 2000; Waters & Cummings, 2000). Indicators of competence include more positive self-esteem, stronger self-regulation, better interpersonal skills, higher levels of educational attainment, and more effective parenting in the next generation (Masten & Coatworth, 1998; Simpson et al., 2007; Sroufe et al., 2005).

Currently, most of the research that assesses the emotional atmosphere of individuals’ families-of-origin does so by either examining emotional qualities exclusively within parent–child dyads (e.g., via assessing attachment bonds, expressed emotion, or relationship quality, Peris & Miklowitz, 2015; River et al., 2022; Sroufe et al., 2005) or positive relationships including, but not limited to, those that occur within the family (e.g.,Bethell et al., 2019; Narayan et al., 2018). Less research, however, has focused on assessing emotional connectedness within the family exclusively but more broadly than between just the parent–child dyad. This includes the emotional connectedness between individuals and their parents/primary caregivers, as well as across household members according to a target individual’s perspective. This study introduces a novel concept, Centeredness, which assesses the emotional bonds and connectedness between target individuals and their primary caregivers (biological parents or adults primarily responsible for caregiving, herein after referred to as “parents,”), and the general emotional atmosphere within the primary childhood home environment, including with other family members.

This study had two primary goals. The first goal was to develop an instrument that could be used with adult respondents about their childhood perceptions of feeling emotionally supported, connected, accepted, and validated within their families. It was developed to apply to many diverse family structures and compositions, including those not necessarily characterized by two biological parents at the head of the household. Instruments that assess family-of-origin relationships through a diverse and inclusive lens are scarce (Bell & Bell, 2018; Causadias & Cicchetti, 2018). Relatedly, a secondary goal was to create an instrument that could easily be used in more practical and clinical settings to help current adults understand how to bring forth this same degree of emotional connectedness with their own children. Research on the intergenerational transmission of positive caregiving continually suggests that having concrete templates of positive parenting from one’s childhood are a critical step in transmitting loving and supportive caregiving into the next generation (Lieberman et al., 2005; Narayan et al., 2020). Just as many existing instruments on positive and supportive parent–child or family environments do not lend themselves to diverse family structures, many instruments also do not allow adults who are parents to gain insight while completing the instrument itself about how to bring forth similar positive experiences with children. To address these goals, this study introduced a novel construct and instrument to assess Centeredness.

## Theoretical Definitions of Centeredness and Related Constructs

### Theoretical Definitions

**Fig. 1:**
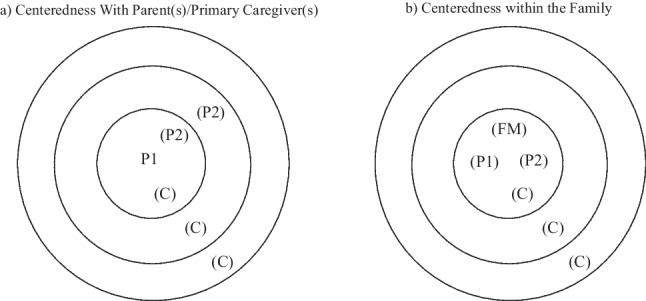
Conceptual diagram of childhood Centeredness (**a**) with parents and (**b**) within the family. Please note: P1, parent or primary caregiver 1; P2, second parent or secondary caregiver; C, child (target respondent); FM, any other family member(s) in household (e.g., siblings, grandparents, etc.); one or more FM(s) may be present in the right diagram. Parentheses indicate various possible positions that could relate to the child’s different perceptions of overall Centeredness. The left circle is the most common case of having a parent (P) at the center of the family. In this case, it is a two-parent family, but P2 could be removed for a single-parent family. The child (C) could be in one of three places, corresponding to high perceived overall Centeredness (innermost circle), moderate Centeredness (second tier), or low Centeredness (outermost circle). The right circle adds in other family members (FMs) that might live in the primary household. If everyone is in the inner circle, then the child (C) would perceive high overall Centeredness within the family, but as the child or any other members move outward, the child’s overall Centeredness score would theoretically decrease as the child perceives themself or others to not be as centered in the family environment. To receive a high score on the Centeredness scale, a target adult respondent would have to perceive high levels of overall Centeredness in relation to their parent(s)/primary caregiver(s) (left diagram) and in relation to their entire family unit ((right diagram). Target adult respondents who perceived high Centeredness in only one or the other (or neither)  diagram would theoretically have lower overall Centeredness scores on the Centeredness scale. Finally, perceptions of Centeredness may vary across different family members, such that two siblings in the same family may perceive different levels of Centeredness (and have different overall scores on the Centeredness scale)

Centeredness is defined as the perception of belonging and feeling emotionally connected and unconditionally accepted and supported within the childhood family of origin. The term Centeredness originated from clinical observations of having a child place themselves, their parents, and any other family members in their household within a series of concentric circles intended to represent various degrees of closeness and connectedness (i.e., Centeredness) within a family. The child was instructed to place themselves and all members closer to or further from the innermost circle according to the extent to which they perceived themselves and other family members to be close and connected to each other (see Fig. 1). Anecdotally, children were observed to typically place their parents (usually but not always the biological mother and/or father) in the center. Some children would put themselves close to that person/people, while other children would put themselves farther out towards the outer edges of the circles. We then observed that children who placed themselves on the outer edges of the circle tended to show more adjustment problems (e.g., sadness, worry, and anger) than children who placed themselves closer to the center, prompting us to wonder whether the effects of feeling “centered” during one’s childhood would continue into adulthood.

### Related Theoretical Foundations

The concept of Centeredness also integrates components from several well-established contemporary and historical theories, including attachment theory, ecological systems theory (EST), and belongingness theory (Bronfenbrenner & Morris, 1998; Baumeister & Leary, 1995; Masten, 2006; Sroufe et al., 2005). Childhood attachment theory posits that primary caregivers’ sensitivity and responsiveness to infants’ emotions and exploration shape infants’ expectations that social partners are supportive, trustworthy, and capable of meeting emotional needs (Sroufe, 2020; Waters & Cummings, 2000). Centeredness also draws from the concept that parents and caregivers are a fundamental source of security and a safe haven for emotional expression (Sroufe et al., 2005; Thompson, 2000). Like attachment theory, Centeredness theory emphasizes the role of primary caregivers in scaffolding emotional development, but it also assumes that, in addition to the parent–child dyad, other family members (e.g., relatives living in the household and siblings) shape the household emotional climate and individuals’ perceptions of safety and security within the family.

Centeredness also echoes EST, which views the developing child as encompassed within a nested, translational set of systems, including the family, broader community, and society (Bronfenbrenner & Morris, 1998). Centeredness similarly places the child within a set of nested, transactional relationships. Like EST, Centeredness theory presumes that interacting relationship systems that do not directly include the child (e.g., parent-sibling relationships and the interparental relationship) may still affect a child’s emotional wellbeing and perceptions of acceptance and belongingness (Bronfenbrenner & Morris, 1998; Masten, 2006). Unlike EST, however, Centeredness focuses more deeply and exclusively on relationships and emotional bonds within the primary or most formative family-of-origin household, rather than on broader socioecological relationships outside the family (Bronfenbrenner & Morris, 1998).

Finally, belongingness theory involves individuals’ need to perceive companionship, affiliation, and connectedness to key social groups, including the broader societal context (Baumeister & Leary, 1995; Hagerty & Patusky, 1995; Malone et al., 2012). Centeredness draws on these assumptions but specifically focuses on individuals’ needs to make and preserve deep and secure emotional bonds and feel valued and accepted within the family of origin, as opposed to more broadly across individuals’ interpersonal networks. Centeredness applies more narrowly to everyone whom the individual considers to be members of their primary childhood household.

The current concept of Centeredness also echoes the recent focus in the last five years on the role of positive childhood experiences within the public health literature. More specifically, this body of research has identified positive experiences in childhood as important influences that counteract the effects of childhood adversity and promote better long-term health and wellbeing across generations (CDC, 2021; Hays-Grudo et al., 2021; Narayan et al., 2021). While public health research has shown that higher levels of adults’ adverse childhood experiences (ACEs) predict higher levels of mental and physical health problems, greater suicide risk, and earlier morbidity (Dube et al., 2001; Felitti et al., 1998), recent attention has shifted to the role of positive childhood experiences within this field. This research has shown that adults’ positive childhood experiences, such as safe, supportive relationships and a positive, predictable quality of life, may reduce the negative consequences of ACEs on health outcomes through independent health-promoting pathways or as direct buffers against ACEs (Narayan et al., 2021). Recent work has pointed to the importance of positive *relational* experiences of childhood more specifically, including perceptions of support and belongingness from family, friends, the school, and the community, in predicting better adulthood mental health in population-based samples (Bethell et al., 2019).

Taking together, historical literature has emphasized the importance of children’s healthy and nurturing relationships within their families (Masten, 2001; Sroufe et al., 2005), and recent literature has focused on the role positive childhood experiences in the context of childhood adversity (Bethell et al., Narayan et al., 2018). However, these literatures still lack efficient, inclusive tools to capture the emotional connectedness within families specifically, including those of diverse structure and composition. While several instruments index qualities of warm, supportive family environments and attachment relationships, none of these instruments assess supportive, accepting, and validating home environments specifically within families of diverse backgrounds who may have grown up in non-traditional households.

## Centeredness Instrument Development and Related Instruments

### Centeredness Instrument Development

Based on the clinical observations described above and existing theoretical foundations, we created an instrument developed to capture a target adult’s retrospective perceptions of their “Centeredness” within their primary childhood home environment. The original instrument contained 23 total items. For the first 10 items, individuals were asked to “imagine the home where [they] spent the most time as a child, the place that was the most influential or formative for [their] development.” Individuals then responded to questions about how often it was true (using Likert scales of 1—“Never true” to 5—“Very often true”) that all the people who spent the majority of time in that home contributed to an atmosphere in which the individual perceived connectedness, support, and acceptance versus felt like an “outsider,” aligned with the concentric circle concept (see Tables 1 and 2 for exact instructions and all item wording). These first 10 Likert responses were modeled after existing gold-standard instruments on retrospective childhood experiences, such as the Childhood Trauma Questionnaire (Bernstein & Fink, 1998). Next, the individual responded to 13 items about the extent to which they agreed or disagreed with statements about their connectedness to their parents specifically, as well as the perceived support, unconditional acceptance, and emotional validation that they received from their parents. These 13 items were rated on 1–5-point Likert scales ranging from 1—“Strongly disagree” to 5—“Strongly agree.” A final Centeredness score was computed by reverse-scoring indicated items and then summing all items [following psychometric testing (see the “Method” section), three of the 23 items were dropped]. The final Centeredness sum score contains 20 items with possible total scores ranging from 20 (if after reverse scoring, all items are rated as “1”) to 100 (if after reverse scoring, all items are rated as “5”).

**Table 1:** Centeredness items and gender differences for Sample 1

Item *#*	Item wording: Growing up between the ages of 0 and 18…	Male (*n* = 243)	Female (*n* = 293)	Gender non-conforming (*n* = 12)	ANOVA *p* value	Sign. contrast (*p* < .017)
1	After a bad day, I could count on my family to make me feel better.	3.3 (1.1)	3.2 (1.2)	3.3 (1.4)	.98	–
2	I felt like an outsider in my family. (reversed)	3.5 (1.3)	3.1 (1.4)	3.8 (1.1)	.01	M vs. F
3	My home and family were perfect.	2.7 (1.2)	2.3 (1.2)	2.4 (1.2)	.00	M vs. F
4	When I came home at the end of a long day, I expected my home environment to feel tense or unpredictable. (reversed)	3.4 (1.3)	3.3 (1.4)	3.5 (1.4)	.36	–
5	I felt like my emotions were dismissed as incorrect (e.g., “You are overreacting”). (reversed)	3.1 (1.3)	2.6 (1.3)	2.8 (1.0)	< .001	M vs. F
6	My family valued my input.	3.3 (1.1)	3.0 (1.1)	3.7 (1.0)	.01	–
7	I felt unnoticed when I was around my family. (reversed)	3.7 (1.1)	3.6 (1.2)	4.0 (1.1)	.19	–
8	I felt completely satisfied with my home and my family.	3.1 (1.2)	2.9 (1.2)	3.4 (.90)	.01	–
9	I was nervous that someone in my family would say or do something hurtful. (reversed)	3.3 (1.3)	3.1 (1.4)	3.3 (1.1)	.10	–
10	I received enough one-on-one time with my parents.	3.5 (1.2)	3.5 (1.2)	3.5 (1.2)	1.00	–
11	My parents were frequently and easily upset. (reversed)	2.9 (1.2)	2.9 (1.3)	2.7 (1.2)	.79	–
12	When I was upset, I felt like my parents tried to find a way to be on my side.	3.2 (1.1)	3.0 (1.2)	3.3 (1.1)	.11	–
13	My parents believed that I made good choices.	3.5 (1.1)	3.5 (1.2)	3.3 (1.2)	.88	–
14	My parents pointed out positive things about me.	3.7 (1.1)	3.5 (1.2)	4.3 (.8)	.04	–
15	My parents tried to understand how I was feeling by putting themselves in my shoes.	2.8 (1.2)	2.6 (1.2)	2.7 (1.0)	.28	–
16	It would hurt our relationship if I chose different views/beliefs (e.g., religious, political, etc.) than my parents. (reversed)	3.1 (1.3)	2.8 (1.4)	3.3 (1.5)	.02	M vs. F
17	I felt like my parents were happy to see me when I came home after being gone for the day.	3.8 (1.1)	3.6 (1.2)	3.8 (.9)	.39	–
18	When I was upset, I felt like my parents couldn’t handle my negative emotions (e.g., they left the room or told me I shouldn’t feel that way). (reversed)	3.1 (1.3)	2.6 (1.4)	3.1 (1.3)	< .001	M vs. F
19	My parents mentioned ways they were proud of me to other people (e.g., family members, their friends, other adults, etc.).	3.9 (1.1)	3.7 (1.1)	4.1 (1.0)	.33	–
20	My parents had views (religious, political, racial, cultural, etc.) that made me feel hesitant to express in front of others because I didn’t agree with them, or I worried that others would not agree with them. (reversed)	3.3 (1.3)	3.2 (1.4)	3.4 (1.6)	.36	–

Note. Sign. contrast (last column) shows any pairwise contrasts that were significant at *p* < .017 across the three gender groups (*M*, male; *F*, female)

**Table 2:** Factor loadings from the EFA in Sample 1

Item #	CFA item	Item wording: Growing up between the ages of 0 and 18…	EFA loading	Factor
2	f2	I felt like an outsider in my family. (reversed)	.59	1
3	f3	My home and family were perfect.	.71	1
4	f4	When I came home at the end of a long day, I expected my home environment to feel tense or unpredictable. (reversed)	.75	1
8	f8	I felt completely satisfied with my home and my family.	.72	1
9	f9	I was nervous that someone in my family would say or do something hurtful. (reversed)	.67	1
11	p2	*My parents were frequently and easily upset. (reversed)*	*.60*	*1*
12	p3	*When I was upset, I felt like my parents tried to find a way to be on my side.*	*.66*	*2*
13	p4	*My parents believed that I made good choices.*	*.55*	*2*
14	p6	*My parents pointed out positive things about me.*	*.75*	*2*
15	p8	*My parents tried to understand how I was feeling by putting themselves in my shoes.*	*.61*	*2*
19	p12	*My parents mentioned ways they were proud of me to other people (e.g., family members, their friends, other adults, *etc*.).*	*.68*	*2*
10	p1	*I received enough one-on-one time with my parents.*	*.50*	*2*
18	p11	*When I was upset, I felt like my parents couldn’t handle my negative emotions (e.g., they left the room or told me I shouldn’t feel that way). (reversed)*	*.48*	*2*
16	p9	*It would hurt our relationship if I chose different views/beliefs (e.g. religious, political, *etc*.) than my parents. (reversed)*	*.71*	*3*
20	p13	*My parents had views (religious, political, racial, cultural, *etc*.) that made me feel hesitant to express in front of others because I didn't agree with them, or I worried that others would not agree with them. (reversed)*	*.62*	*3*
1	f1	After a bad day, I could count on my family to make me feel better.	.59/.53	1 or 2
6	f6	My family valued my input.	.46/.63	1 or 2
17	p10	*I felt like my parents were happy to see me when I came home after being gone for the day.*	*.47/.59*	*1 or 2*
5	f5	I felt like my emotions were dismissed as incorrect. (reversed)	.45/.48	1 or 2
7	f7	I felt unnoticed when I was around my family. (reversed)	.50/.45	1 or 2
21	f10	If I needed help, I knew I could turn to my family.	.59	Removed
22	p5	*My parents were patient with my normal childhood behaviors (e.g., making a mess while playing, sleeping late, spending a lot of time in my room).*	*.45*	*Removed*
23	p7	*My parents thought mental health problems and going to therapy or counseling were silly or unimportant.*	*.40*	*Removed*

Note. The non-italicized items use the instructions and a five-point scale of “Never true” to “Very often true”: “Please imagine the home where you spent the most time as a child, the place that was the most influential or formative for your development. Now, please also imagine the people that were part of that home. When you read the word “family,” this should include all the people who spent the majority of time in this home.” The italicized items use the instructions and the five-point scale of “Strongly disagree” to “Strongly agree”: Please answer the following questions about your parents (the adults in your home who were responsible for taking care of you while you were growing up) by selecting how much you disagree or agree with the following statements

For example, if the italicized statements were true of only one parent (or neither of your parents), but not both of your parents, then you might agree less with the statements. If items loaded onto two factors, loadings are presented for factor 1, followed by factor 2. A CFA diagram is presented in Fig. 2 in the Supplemental Material

The reasoning for first asking about connectedness to and support and acceptance from family members as a whole and then asking specifically about parents was twofold. The first reason was to create an inclusive measure that does not assume traditional family structures (e.g., two biological parents at the head of the household). The second reason was to guard against the possibility that some individuals may have had a very positive or very negative relationship with one or both parents that was not reflective of the home environment as a whole. Furthermore, participants were asked to respond to items on two different Likert scales with different response formats in order to ensure that participants were not responding in an overly positive or negative manner and were thoughtfully varying their responses based on individual questions. Some items were reverse scored (e.g., “I felt unnoticed around my family”) for this same reason, to guard against response biases. Even though some items on the Centeredness scale are not worded positively, the overall Centeredness sum score reflects a positive construct, with higher scores on Centeredness reflecting more supportive childhood home environments.

### Related Existing Instruments

One of the closest existing instruments to the Centeredness scale is the Experiences in Close Relationships-Relationship Structures (ECR-RS) scale (Fraley et al., 2011). Like the Centeredness scale, the ECR-RS also draws on attachment theory and allows for adult individuals to reflect on their attachment experiences with key caregivers and across close relationships more globally. However, ECR-RS administration directions do not clearly specify how attachment relationships *in childhood* (as opposed to current attachment/relationship representations) would be flexibly and efficiently assessed for individuals from diverse, non-traditional family structures and compositions, including siblings and relatives who lived in the household (Fraley, 2014). Furthermore, ECR-RS item wording does not as easily lend itself to enabling current parents who grew up in unsupportive, invalidating family environments to recognize how they could pivot away from their negative past experiences and strengthen current relationships with children. For instance, ECR-RS items such as “I found it easy to depend on others” and “It helped to turn to others in times of need” do not give current parents a clear sense of how they could implement or strengthen these relational experiences for their children. By contrast, the Centeredness scale can be used with diverse family structures and compositions as it assesses “home” and “family” based on individuals’ unique perceptions of who constituted their most formative childhood household (which often vary among individuals within a study). Further, the Centeredness items could lend themselves to preventive programs to strengthen current family relationships because parents could generate concrete strategies to implement or strengthen these experiences for their children. Centeredness items such as “My parents tried to understand how I was feeling by putting themselves in my shoes,” and “My parents mentioned ways they were proud of me to other people” could be implemented with one’s current children with relative ease.

The Centeredness scale also differs in important ways from other instruments recently developed to assess positive aspects of childhood experiences or relationships. For instance, while the BCEs scale (Narayan et al., 2018) assesses some similar aspects of positive family relationships (e.g., the presence of at least one safe caregiver), it also assesses other non-relational internal resources (e.g., positive core beliefs) as well as positive relationships outside of the home (the presence of at least one close friend and caring teacher). The BCE scale does not assess family relationships deeply or exclusively nor does another recent index of positive childhood experiences that adapted items from the well-validated Child and Youth Resilience Measure (CYRM, Ungar & Liebenberg, 2011). Like BCE findings, findings using this positive childhood experience (PCE) index show that cumulative sets of favorable experiences predict better adulthood adjustment and lower mental health problems (Bethell et al., 2019). Unlike the BCE scale and PCE index, however, the Centeredness scale focuses more deeply on the nuances of the emotional atmosphere and relationships within the primary childhood home environment. The Centeredness scale more closely approximates the *quality* of the family atmosphere, rather than the *quantity* of positive or negative experiences for the better (i.e., BCEs) or the worse (ACEs).

The Centeredness scale also offers advantages over several other validated measures of parent–child relationships and family-of-origin household dynamics, such as the Parental-Acceptance-Rejection Questionnaire (PARQ; Rohner & Khaleque, 2005), and the Relationship Dimensions of the Family Environment Scale (FES; Moos & Moos, 1987). The PARQ assesses similar constructs to the Centeredness scale, such as warmth, emotional, support, and acceptance but does so in regard to traditional dyadic relationships with mothers and fathers. It therefore does not allow for efficient and flexible adaptation to families where individuals were raised by primary caregivers who were not their biological parents (e.g., a grandmother or foster parent).

Like the Centeredness scale, the FES allows for reflection across diverse family structures and compositions. (Indeed, the FES uses terms such as “our family” and “family members” throughout). However, the FES does not allow for target respondents to reflect on their distinct experiences within the family nor to generate concrete strategies for parents to improve relationships with current children. For instance, one of the FES items measuring family cohesion, “We get along well with each other” (Moos & Moos, 1987) applies to family members as a whole. However, it does not specify the target respondent’s role in the dynamic of “getting along,” whom the family members were that got along, if the term “family members” includes everyone in the family or only some members, or how a current parent might go about “getting along” better with their current child. An additional advantage of the Centeredness scale over the above measures (e.g., the PARQ and the FES), is that it is publicly available and free to use, including for non-academic audiences such as clinical practitioners and community providers.

## Childhood Centeredness and Young Adulthood Mental Health

If the Centeredness scale is a valid indicator of the emotional aspects of childhood relationships, which have been deemed possible to validly assess with retrospective instruments (Bell & Bell, 1986), then higher levels of Centeredness should associate with better mental health outcomes and adjustment. In other words, higher levels of Centeredness should be expected to predict similar adulthood outcomes as other validated instruments that assess positive childhood experiences, relationships, and general belongingness. These outcomes include lower levels of internalizing and externalizing problems, such as depression and anxiety symptoms and aggressive behavior; less severe psychological distress, such as fewer suicidal thoughts and behaviors (STBs); and higher levels of life satisfaction (Crandall et al., 2021; Malone et al., 2012; Muraru & Turliuc, 2012; Sroufe et al., 2005).

## The Current Study

This study tested the psychometric properties of the Centeredness scale as a predictor of multiple dimensions of young adults’ mental health and adjustment in two separate samples. All aims and hypotheses were first tested in Sample 1, collected before the pandemic, and examined for replication effects in Sample 2, conducted during the pandemic. Following exploratory factor analysis (in Sample 1) and then confirmatory factor analysis (in Sample 2) to yield a final Centeredness scale, the first aim focused on establishing construct validity. It was hypothesized that in both samples, Centeredness would show good construct validity (i.e., significant associations) with closely-related constructs, attachment-related avoidance and attached-related anxiety, assessed with the ECR-RS. The second aim focused on predictive validity and examined whether Centeredness was associated with young adulthood mental health outcomes over and above attachment-related avoidance and anxiety, and other well-established indices of childhood experiences, total BCEs and ACEs, as well as sociodemographic factors. This aim specifically examined whether the emotional *quality* of the childhood home environment (i.e., Centeredness) predicted mental health outcomes above and beyond the quantity of adverse and positive childhood experiences (i.e., ACEs and BCEs). It was hypothesized that in both samples, higher levels of Centeredness would predict lower levels of depression and anxiety symptoms; suicidal thoughts and behaviors (STBs); and aggressive behavior; and higher levels of life satisfaction. In relation to this aim, it was also expected that the Centeredness scale would outperform the ECR-RS in predicting lower levels of these mental health problems and a dimensional composite of outcomes when all other predictors (including BCEs, ACEs, and sociodemographic factors) were considered together.

## Method

Sample 1 included 548 participants (*M* = 27.1 years, *SD* = 4.6, range = 19–35 years; 53.5% female, 44.3% male, 2.2% gender non-conforming, 68.3% White, 9.5% Black, 8.9% Asian, 8.2% Latine, 4.6% biracial, multiracial, or other, and 0.5% prefer not to respond), and Sample 2 included 1198 participants (*M* = 26.4 years, *SD* = 4.8, range = 19–35 years; 56.2% female, 41.5% male, 2.3% gender non-conforming, 66.4% White, 10.9% Asian, 8.5% Latine, 8.3% Black, 5.7% biracial/multiracial/other, and 0.2% prefer not to respond). All participants were drawn from a larger two-sample study on the associations between childhood experiences and relationships and young adulthood outcomes with the purpose of collecting two large samples for hypothesis testing and replication. These studies were conducted via REDCap, the secure, web-based platform for data collection and management (Harris et al., 2019), and Prolific-Academic (Pro-A), an online crowd-sourcing platform that produces high-quality social and behavioral empirical data, including on retrospective instruments assessing childhood experiences (Eyal et al., 2021; Green & Douglas, 2018; Peer et al., 2017).

Participants were recruited through Pro-A and were eligible if they were 19 to 35 years old, spoke English, and were born in and currently lived in the USA. Participants were also required to have a Pro-A approval rating of at least 99%, a criterion provided by Pro-A to confirm that participants have a successful survey completion and quality rating measured by valid, timely, and complete survey submissions. Eligible participants received REDCap links to complete online informed consent and a 1-hour survey, which was composed of publicly available, well-validated standardized questionnaires. Participants in Sample 1 completed the study between November 12, 2019, and January 19, 2020 (before the COVID-19 pandemic), and participants in Sample 2 completed the study between August 18, 2020, and December 1, 2020 (during the COVID-19 pandemic and over the 2020 US election period). All participants were compensated, given debriefing information, and provided with links for mental health referrals.

## Measures: Childhood Predictors/Independent Variables

### The Centeredness Scale: Item Development

As described in the introduction, the Centeredness scale was developed as a novel instrument to expand existing measures of positive childhood experiences and relationships within diverse households and family compositions. Items were developed according to the following theoretically- and empirically supported assumptions: (1) attachment figures are a safe haven for children to express the full range of emotions; (2) one’s primary childhood household represents an emotionally safe environment from which to recover from the stressors and difficulties of daily life; (3) the family unit provides a source of connectedness and belonging from which children feel like valued members and not like outsiders; and (4) family members, inclusively defined, accept children for their unique identities and core beliefs (Bronfenbrenner & Morris, 1998; Masten, 2018; Moos & Moos, 1987; Sroufe et al., 2005). Initially, 23 items were created with careful attention to wording that could easily lend itself to translation efforts to strengthen existing family relationships. All 23 items were subjected to exploratory factor analysis (EFA) in Sample 1 and confirmatory factor analysis (CFA) in Sample 2 to confirm that the items cohered into one overall construct.

#### Exploratory Factor Analysis (Sample 1)

We first used EFA techniques with Sample 1 to examine the total possible number of latent variables (factors). We used the R packages “psych” and “nfactors” (Revelle & Revelle, 2022), including psych’s “fa.parallel” algorithm that creates 1000 simulations and finds the number of factors (illustrated by eigenvalues) that are above the background simulation value. We selected the threshold of ≥ .45 for each item’s eigenvalue to load on any detectable factor. Various authors over the years have made arguments for different cut-off values, and a common value is .40 (Stevens, 2012). We decided to use .45 because it was slightly more conservative and allowed for exclusion of some of the items with weaker loadings (see below).

We used “nfactors” to estimate the number of factors based upon Velicer MAP and BIC metrics. These techniques produced a value for the likely number of factors. We then used psych’s “fa” function to generate the loading of each item onto each of the proposed factors. We selected varimax rotation to most clearly detect the possible loadings of items onto final factors because this option makes the factors orthogonal to each other (Kaiser, 1958). After detecting the plausible number of factors and the item-to-factor loadings, we used CFA to test the model with Sample 2. For testing, we fitted a CFA model using the R package “lavaan” (Rosseel, 2012).

Results from the EFA in Sample 1 revealed three factors (i.e., fa.parallels, MAP, and BIC all showed 3 factors). Table 2 shows the loading of individual items onto the proposed three factors from Sample 1. These results show clear separation for most items into three factors with the majority of the items loading predominantly onto factors 1 and 2. Upon inspection of the final items and their question-to-factor loadings (Table 2), three items were excluded from the Centeredness scale for the following reasons. One item’s wording (#21, f10, Table 2) was redundant with another item (#1, f1), and both had comparable loadings (.59), so the former was eliminated due to having less clear wording. Another item’s wording was also unclear and its loading barely surpassed our threshold (#22, p5, .45). Finally, a third item (#23) did not load onto any of the three factors according to our threshold (#23, p7, .40).

#### Confirmatory Factor Analysis (Sample 2)

After removing the three aforementioned items, we created a CFA model with the 20 remaining outcomes and tested it in Sample 2 (see Fig. 2 in Supplemental Material). Of these 20 items, nine items pertained to the home environment/family and 11 pertained to parents/parental figures. Of the 20 items, 18 items loaded onto one of two factors (with some loading comparably on both; see Table 2), and two items loaded onto a third factor (Table 2). This model significantly accounted for the data (*p* < .0001) and produced model metrics that are considered adequate but not necessarily good [Comparative Fit Index (CFI) = .91, Tucker-Lewis Index (TLI) = .90], as thresholds for “good fit” tend to be CFI ≥ 0.95 (Hooper et al., 2008). Because these results did not provide clear or convincing evidence that there are indeed three distinct factors within the Centeredness scale (because model fit characteristics were adequate but not good) and because we did not set out to identify subfactors or subscales of Centeredness, we retained all 20 items (Table 1). A total Centeredness score was then computed in each sample by reverse-scoring indicated items and then summing all items (Sample 1: *M* = 63.9, *SD* = 17.5, range = 20–100, *α* = .95; Sample 2: *M* = 65.8, *SD* = 17.5, range = 20–100,* α* = .95).

### Childhood Attachment-Related Avoidance and Anxiety

Participants completed the ECR-RS scale, consisting of nine items (four reverse-coded) assessing attachment anxiety and avoidance with their mothers (or mother figures) and fathers (or father figures), respectively. The ECR-RS has strong psychometric properties and excellent test–retest stability (*r* = .80) for attachment representations with parents (Fraley et al., 2011). Together, the nine items showed good internal consistency regarding attachment representations with both parents in the present study (Sample 1: *α*_mother_ = .93, *α*_father_ = .93; Sample 2: *α*_mother_ = .93, *α*_father_ = .93). As recommended by the developers, the current study used one score averaged across responses for mothers and fathers for attachment-related avoidance and a separate score averaged across mothers and fathers for attachment-related anxiety (Sample 1 attachment avoidance: *M* = 3.9, *SD* = 1.4, range = 1–7, and attachment anxiety: *M* = 2.5, *SD* = 1.5, range = 1–7; Sample 2 attachment avoidance: *M* = 3.7, *SD* = 1.5, range = 1–7, and attachment anxiety: *M* = 2.4, *SD* = 1.4, range = 1–7). Though some research has used this instrument to assess *current* global attachment representations relationships (Gidhagen et al., 2021), we did not modify the original ECR-RS instrument to assess *childhood* global attachment-related avoidance and anxiety (e.g., across all household family members) because the ECR-RS does not provide instructions nor psychometric information about such adaptations (Fraley, 2014).

### Benevolent Childhood Experiences (BCEs)

Participants completed the BCEs scale, which has good psychometric properties and high test–retest stability (*r* = .80; Narayan et al., 2018). It consists of 10 items assessing the presence of childhood relationships and resources and a positive and predictable quality of life that have been found to be culturally generalizable. Responses were summed in both studies for one total BCEs score (Sample 1: *M* = 7.6, *SD* = 2.1, range = 1–10; Sample 2: *M* = 7.8, *SD* = 2.2, range = 1–10).

### Childhood Adversity

Participants completed the 10-item ACE scale (CDC, 2021; Felitti, 1998), which has high test–retest stability (*r* = .79) and good convergent validity with other instruments assessing childhood adversity (Schmidt et al., 2020). The ACE scale consists of five items assessing childhood maltreatment (i.e., emotional, physical, and sexual abuse, and physical and emotional neglect) and five items assessing childhood exposure to family/household dysfunction (i.e., exposure to parental separation/divorce and domestic violence, parental/household member mental illness, substance use, and incarceration) between the ages of 0 and 18 (see Schmidt et al., 2020 for full and exact item wording). Responses were summed in both studies for one total childhood adversity score (Sample 1: *M* = 2.3, *SD* = 2.2, range = 0–10; Sample 2: *M* = 2.0, *SD* = 2.2, range = 0–10). Although the continuous total score was used in analyses, ACEs were prevalent: in Sample 1, 71.2% of participants reported at least one ACE, and 25.5% reported four or more ACEs, a threshold in the literature that has associated with multiplicative risk for adulthood health problems (Dube et al., 2003). In Sample 2, 62.2% reported at least one ACE, and 19.9% reported four or more ACEs.

## Measures: Young Adulthood Mental Health Outcomes/Dependent Variables

### Depression Symptoms

Participants completed the Patient Health Questionnaire (PHQ-9; Spitzer et al., 1999), a nine-item, self-report checklist assessing severity of depression symptoms in the past 2 weeks on four-point Likert scales (1—“Not at all” to 3—“Nearly every day”). The PHQ-9 has excellent internal consistency (*α* = .86–.89) and test–retest reliability (*r* = .84; Kroenke et al., 2001). A total score was created by summing all responses (Sample 1: *M* = 9.0, *SD* = 6.9, range = 0–27, *α* = .90; Sample 2: *M* = 8.8, *SD* = 6.8, range = 0–27, *α* = .90). Although this study used continuous symptom scores, both samples reported elevated rates of clinical depression according to the cutoff score ≥ 10 recommended by the developers (Kroenke et al., 2001). In Sample 1, 39.6% of participants reported clinical levels of depression, and in Sample 2, 40.7% of participants reported clinical depression.

### Suicidal Thoughts and Behaviors (STBs)

Two items were composited for a variable reflecting STBs, operationalized as a history of a lifetime suicide attempt (“Have you ever made a suicide attempt” (Sample 1: 25.9%; Sample 2: 25.2%) and current thoughts of death or self-harm (i.e., the ninth item on the PHQ-9, “[In the past two weeks] thoughts you would be better off dead or hurting yourself in some way,” collapsed across response options, 1—“Several days” to 3—“Nearly every day” Sample 1: 15.5%; Sample 2: 13.4%). These two items were summed in both samples for one 0–2-point variable reflecting STBs (Sample 1: *M* = 0.4, *SD* = 0.7, range = 0–2; Sample 2: *M* = 0.4, *SD* = 0.6, range = 0–2).

### Anxiety Symptoms

Participants completed the Generalized Anxiety Disorder scale (GAD-7), a seven-item self-report checklist assessing the severity of anxiety symptoms in the past 2 weeks on four-point Likert scales (0—“Not at all” to 3—“Nearly every day”). The GAD-7 has high internal consistency (*α* = 0.93) and test–retest reliability (*r* = .83; Spitzer et al., 2006). A total score was created by summing all responses (Sample 1: *M* = 7.5, *SD* = 5.9, range = 0–21, *α* = .92; Sample 2: *M* = 7.3, *SD* = 6.0, range = 0–27, *α* = .92). Although this study used continuous symptom scores, both samples reported elevated rates of clinical anxiety according to the cutoff score ≥ 10 recommended by the developers (Spitzer et al., 2006). Using this cutoff in Sample 1, approximately 32.3% of participants reported clinical levels of anxiety, and in Sample 2, 31.3% of participants reported clinical anxiety.

### Aggressive Behavior

Participants completed the short form of the Buss-Perry Aggression Questionnaire (BPAQ-SF; Bryant & Smith, 2001; Buss & Perry, 1992;), a 12-item scale that assesses individuals’ use of physical and verbal aggression, and expressions of anger and hostility on five-point Likert scales of (1—“Extremely uncharacteristic of me” to 5—“Extremely characteristic of me”). This scale has good internal consistency (*α* = .70–.83; Bryant & Smith, 2001), and excellent test–retest reliability (*r* = .80; Buss & Perry, 1992). This study used a continuous score of the average of the 12 items (Sample 1: *M* = 2.6, *SD* = 0.7, range = 1.2–4.8, *α* = .77; Sample 2: *M* = 2.5, *SD* = 0.7, range = 1.0–4.8, *α* = .81), with one item (“I am an even-tempered person”) reverse-coded.

### Life Satisfaction

Participants completed the Satisfaction with Life Scale (SWLS; Pavot & Diener, 1993) a five-item scale that assesses individuals’ current quality of life according to personal standards. Items are rated on seven-point Likert scales from 1—“Strongly disagree” to 7—“Strongly agree;” and examples include, “I am satisfied with my life” and “In most ways, my life is close to ideal.” The SWLS has high internal consistency (*α* = .87) and test–retest reliability (*r* = .82; Pavot & Diener, 1993). This study used a continuous total score for life satisfaction (Sample 1: *M* = 19.1, *SD* = 8.2, range = 5–35, *α* = .91; Sample 2: *M* = 20.8, *SD* = 7.8, range = 5–35, *α* = .90). Participants in both studies reported mean levels of life satisfaction consistent with other normative samples of young adults (Pavot & Diener, 1993).

### Dimensional Mental Health Composite

In both samples, a total dimensional composite of all mental health outcomes was created by *z*-scoring the continuous scores for each of the five mental health variables (with life satisfaction reverse-scored) and then averaging the *z*-scores.

## Covariates

In both samples, covariates included participants’ age, gender identity, racial/ethnic identity, current romantic partner status (partnered versus not), current parental status (currently a biological parent or not), educational attainment, and childhood socioeconomic status (SES). All of these covariates were selected because of their theoretical and empirical associations with young adults’ mental health (Masten, 2018; Sayyah et al., 2022; Sroufe et al., 2005). Participants used the following published Likert scales for educational attainment (on a six-point scale): “Less than a high school degree; high school degree or equivalent; some college; associates’ degree; bachelors’ degree; or other higher degree (including M.A., Ph.D., J.D., etc.);” and for childhood income level (on a five-point scale in regard to the household where they spent the most time): “Poor, low-income, middle-class, well-to-do, or wealthy” (Sayyah et al., 2022). See Table 3 for descriptive statistics.

**Table 3:** Descriptive statistics for Sample 1 and Sample 2

	Sample 1			Sample 2			Both Samples
Variable	*M* or %	SD	Sample range	*M* or %	SD	Sample range	Possible range
Centeredness	63.9	17.5	20–100	65.8	17.5	2–100	20–100
ACEs	2.3	2.2	0–10	2.0	2.2	0–10	0–10
BCEs	7.6	2.1	1–10	7.8	2.2	1–10	0–10
Avoidance	3.9	1.4	1–7	3.7	1.5	1–7	1–7
Anxiety	2.5	1.5	1–7	2.4	1.4	1–7	1–7
Age	27.1	4.6	19–35	26.4	4.8	19–35	19–35
Male gender	44.3%	-	-	41.5%	-	-	0–100%
Female gender	53.5%	-	-	56.2%	-	-	0–100%
Non-binary gender	2.2%	-	-	2.3%	-	-	0–100%
White	68.3%	-	-	66.4%	-	-	0–100%
Non-White minority	31.2%	-	-	33.4%	-	-	0–100%
Black	9.5%	-	-	8.3%	-	-	0–100%
Asian	8.9%	-	-	10.9%	-	-	0–100%
Latine	8.2%	-	-	8.5%	-	-	0–100%
Parent (yes)	15.5%	-	-	19.7%	-	-	0–100%
Partnered (yes)	54.6%	-	-	58.9%	-	-	0–100%
Educational attain	4.0	1.4	1–6	4.1	1.3	1–6	1–6
Childhood income	2.8	0.8	1–5	2.9	0.8	1–5	1–5
Depression symptoms	9.0	6.9	0–27	8.8	6.8	0–27	0–27
STBs	0.4	0.6	0–2	0.4	0.6	0–2	0–2
Anxiety symptoms	7.5	5.9	0–21	7.3	5.9	0–21	0–21
Aggressive behaviors	2.6	0.7	1.2–4.8	2.5	0.7	1–4.8	1–5
Life satisfaction	19.1	8.2	5–35	20.8	7.8	5–35	5–35

## Data Analytic Plan and Missing Data

All hypotheses were first examined in Sample 1 and tested for replicability in Sample 2. To address the first aim regarding construct validity, bivariate correlations were conducted between Centeredness and (a) attachment-related avoidance and (b) attachment-related anxiety. Mean levels of Centeredness were also examined across the three gender groups and across the four racial/ethnic groups that had the largest cell sizes in both samples (White, Black, Latinx, and Asian). To address the second aim regarding predictive validity, linear regressions were conducted for the five mental health outcomes and the composite.

All predictors and continuously distributed covariates were entered as *z*-scores in each regression. In anticipation that Centeredness may be significantly associated with other childhood predictors, which would also be intercorrelated, we examined the variance inflation factor (VIF) index of multicollinearity and considered a value ≤ 10 to indicate acceptable collinearity between independent variables (Akinwande et al., 2015). Based on all VIF indices below 10 and the observation that all predictors were correlated at absolute values below cutoffs deemed to be problematic for collinearity (0.80–0.90; Mason & Perreault, 1991), all predictors were entered into each regression together to minimize multiple testing and type 1 error. All predictors and covariates that were dimensional variables were *z*-scored prior to being entered in regressions, while all categorical covariates were entered as dichotomous variables (i.e., non-White minority, romantic partner status, and partner status) or as dummy-coded variables (i.e., gender identity). Given the goals to test and replicate the validity of Centeredness, the “Results” section largely focuses on findings that were significant in Sample 1 and replicated in Sample 2, though some sample discrepancies are noted. All other results are available in the Supplementary Material. All data analysis was performed with SPSS version 25 and R. All data and analysis code for this study are available upon request by emailing the corresponding author.

The overall level of missing data was extremely low. Attrition for Sample 1 was 1.8%, and attrition for Sample 2 was 4.7%, reflecting participants who did not finish the full survey because they stopped before completing all measures. Missing data on individual variables (e.g., because a participant elected to skip a question) in Sample 1 ranged from 0% (covariates) to 5.3% (BCEs), and missing data in Sample 2 ranged from 0% (covariates) to 6.4% (aggressive behavior), with slightly higher rates of missing data on instruments that appeared later in the survey protocol. If participants skipped a question that was part of a composite variable (e.g., total BCEs, overall aggressive behavior, etc.), then that variable was missing for that participant. Across the entire datasets, missing data for Sample 1 was 1.8%, and missing data for Sample 2 was 2.6%, so analyses for missingness were deemed unnecessary.

## Results

### Descriptive Statistics and Sample Comparisons

See Table 3 for descriptive statistics of all variables in both samples. Sample 2 was expected to have higher levels of mental health problems because those participants completed the study during the COVID-19 pandemic and the 2020 US election. However, mean differences on all mental health outcomes between Sample 1 and Sample 2 revealed no differences in any outcome except that participants from Sample 2 reported significantly higher levels of life satisfaction, *t*(1,010) =  − 4.14, *p* < .001, Sample 1: *M* = 19.1; Sample 2: *M* = 20.8.

In terms of additional descriptive statistics, mean levels of Centeredness did not significantly differ between the four major racial/ethnic groups in either sample [Sample 1: *F*(3, 510) = 1.2, *p* = .32; Sample 2: *F*(3, 1104) = 2.4, *p* = .07)]. However, in both samples, mean levels of Centeredness did differ between the three gender groups [Sample 1: *F*(2, 539) = 4.3, *p* = 0.01; Sample 2: *F*(2, 1,174) = 24.3, *p* < .001)]. Post hoc analyses revealed that males in both samples reported significantly higher means on Centeredness (Sample 1: M = 66.1, *SD* = 16.8; Sample 2: M = 69.7, *SD* = 17.0) than females (Sample 1: M = 61.8, *SD* = 17.9; Sample 2 M: = 63.4, *SD* = 17.4) and individuals identifying as gender non-conforming (Sample 1: M = 66.1, *SD* = 16.8; Sample 2: M = 55.4, *SD* = 13.9). Compared to individuals identifying as gender non-conforming, individuals identifying as female reported higher mean levels of overall Centeredness in Sample 2 only). We also examined mean differences in all Centeredness items across all three gender groups using ANOVA tests with pairwise contrasts conducted for any significant ANOVAs. All pairwise contrasts used Bonferroni-corrected *p* values (*α’* = .05/3 = .017, so *p* < .017). Results showed that for five items in Sample 1, individuals identifying as male reported higher scores than individuals identifying as female (see Table 1). We also examined mean differences in all items in Sample 2 (see Table 8, Supplemental Material), which showed more significant pairwise contrasts between all three gender groups than Sample 1 (which may have been due in part the larger size of Sample 2).

For the regressions, all three gender groups were entered as covariates (with male gender dummy coded). Given that mean levels of Centeredness did not differ in either sample across the four racial/ethnic groups with the largest cell sizes, the majority of all participants in both samples identified as White, and the goal to preserve case-wise data across as many participants as possible, racial/ethnic identity was collapsed into White versus non-White minority for the linear regression analyses. However, we acknowledge that this decision may have limited ability to detect differences in mental health outcomes across individuals with different racial/ethnic identities (which was not the focus of this study).

### Aim 1: Construct Validity of Centeredness

Higher levels of Centeredness were significantly associated with higher levels of BCEs in both samples (Sample 1: *r* = .56, *p* < .001; Sample 2: *r* = .61, *p* < .001), as well as lower levels of attachment-related avoidance (Sample 1: *r* =  − .75, *p* < .001; Sample 2: *r* =  − .78, *p* < .001) and attached-related anxiety (Sample 1: *r* =  − .62, *p* < .001; Sample 2: *r* =  − .67, *p* < .001). Additionally, higher levels of Centeredness were significantly associated with lower levels of ACEs in both samples (Sample 1: *r* =  − .66, *p* < .001; Sample 2: *r* =  − .66, *p* < .001).

### Aim 2: Predictive Validity of Centeredness

Bivariate correlations were first conducted between Centeredness and all five mental health outcomes (Table 4 and Tables 9, 10, and 11 in Supplemental Material). In both samples, higher levels of Centeredness were significantly associated with lower levels of depression symptoms (Sample 1: *r* =  − .34, *p* < .001; Sample 2: *r* =  − .33, *p* < .001), STBs (Sample 1: *r* =  − .42, *p* < .001; Sample 2; *r* =  − .42, *p* < .001), anxiety symptoms (Sample 1: *r* =  − .44, *p* < .001; Sample 2: *r* =  − .45, *p* < .001), and aggressive behavior (Sample 1: *r* =  − .34, *p* < .001; Sample 2: *r* =  − .29, *p* < .001); higher levels of life satisfaction (Sample 1: *r* = .43, *p* < .001; Sample 2: *r* = .44, *p* < .001), and lower levels of the dimensional composite (Sample 1: *r* =  − .52, *p* < .001; Sample 2: *r* =  − .53, *p* < .001). Because males reported significantly higher levels of Centeredness in both samples, we conducted correlations between Centeredness and mental health outcomes for males versus females (cell sizes for gender non-conforming individuals were too small) to examine whether Centeredness only associated with mental health outcomes for males. For males and females in both samples, however, higher levels of Centeredness were significantly associated with all mental health outcomes in the same directions described above (all *p* values < .001).

**Table 4:** Bivariate correlations between childhood predictors and mental health outcomes in Sample 1

Independent variables (Childhood predictors)	1	2	3	4	5	6	7	8	9	10	11
1. Centeredness	-	.00	.00	.00	.00	.00	.00	.00	.00	.00	.00
2. ACEs	− .66	-	.00	.00	.00	.00	.00	.00	.00	.00	.00
3. BCEs	.56	− .47	-	.00	.00	.00	.00	.00	.00	.00	.00
4. Avoidance	− .75	.53	− .49	-	.00	.00	.00	.00	.00	.00	.00
5. Anxiety	− .62	.61	− .42	.61	-	.00	.00	.00	.00	.00	.00
Dependent variables (Mental health outcomes)											
6. Depression symptoms	− .34	.33	− .35	.27	.29	-	.00	.00	.00	.00	.00
7. STBs	− .42	.40	− .38	.35	.34	.63	-	.00	.00	.00	.00
8. Anxiety symptoms	− .44	.39	− .35	.31	.29	.78	.49	-	.00	.00	.00
9. Aggressive behavior	− .34	.29	− .22	.25	.27	.34	.28	.29	-	.00	.00
10. Life satisfaction	.43	− .31	.43	− .41	− .29	− .54	− .40	− .43	− .32	-	.00
11. Overall composite	− .52	.43	− .49	.43	.35	.90	.62	.83	.41	− .81	-

Note. Exact correlation coefficients are shown below the diagonal, and exact *p* values are shown above the diagonal. *STBs*, suicidal thoughts and behaviors

Linear regressions revealed that, after controlling for covariates, higher levels of Centeredness significantly predicted all five outcomes and the dimensional composite (six possible outcomes) in the expected direction (negative associations for all outcomes except life satisfaction) in Sample 1, and all six findings replicated in Sample 2 (see Tables 5, 6, and 7 for all regressions for Sample 1, see Tables 12, 13, and 14 in Supplemental Material for all regressions for Sample 2). Higher levels of BCEs also significantly predicted all five outcomes and the dimensional composite in the expected direction in both samples, except for aggressive behavior in Sample 1. Centeredness was therefore a significant predictor of 12 out of the 12 possible outcomes (six outcomes in each of the two samples), and BCEs were a significantly predictor of 11 out of 12 of the possible outcomes. ACEs and attachment-related avoidance or anxiety did not significantly predict as many outcomes in Sample 1. Furthermore, most of these significant findings did not consistently replicate in Sample 2. For instance, ACEs only predicted three of the six possible outcomes (i.e., depression and anxiety symptoms and STBs) in Sample 1 and only replicated in predicting two out of three of these outcomes in Sample 2 (i.e., depression and anxiety symptoms). Attachment-related avoidance only predicted one of the six possible outcomes in Sample 1 (life satisfaction) but this effect did not replicate in Sample 2. Attachment-related anxiety did not significantly predict any of the possible outcomes in Sample 1 (see Tables 5, 6, and 7 and Tables 12, 13, and 14 in Supplemental Material). While these findings indicate that Centeredness predicted a broader number of mental health outcomes than the other predictors (with BCEs a close second), it is important to note that the vast majority of significant findings for any predictor (Centeredness or any of the others) were comparable in magnitude, and most predictors showed a medium effect size (i.e., .15 ≤ *F*^2^ ≤ .35; Cohen, 1988). Therefore, Centeredness generally did not predict outcomes more strongly than the other predictors.

**Table 5:** Linear regression of depression symptoms and STBs on Centeredness and other variables in Sample 1

	Depression symptoms						Suicidal thoughts and behaviors (STBs)					
Variable	*ß*	SE	*t*-value	*p*-value	*F*^2^	*F*^2^ 95% CI	*ß*	SE	*t*-value	*p*-value	*F*^2^	*F*^2^ 95% CI
(Intercept)	9.17	0.54	16.85	.00	.03	[-.13, .18]	0.37	0.05	7.06	.00	-.06	[-.23, .10]
**Centeredness**	** − 0.10**	**0.03**	** − 3.62**	**.00**	** − .25**	**[− .39, − .12]**	** − 0.01**	**0.00**	** − 2.12**	**.03**	** − .16**	**[− .30, − .01]**
**ACEs**	**0.37**	**0.18**	**2.03**	**.04**	**.12**	**[ .00, .23]**	**0.05**	**0.02**	**2.62**	**.01**	**.16**	**[ .04, .27]**
**BCEs**	** − 0.81**	**0.16**	** − 5.07**	**.00**	** − .25**	**[− .34, − .15]**	** − 0.07**	**0.02**	** − 4.28**	**.00**	** − .22**	**[− .32, − .12]**
Avoidance	− 0.21	0.31	− 0.69	.49	− .04	[− .17, .08]	− 0.02	0.03	− 0.51	.61	− .03	[− .17, .10]
Anxiety	− 0.13	0.26	− 0.49	.62	− .03	[− .13, .08]	0.04	0.02	1.55	.12	.09	[− .02, .20]
Age	0.05	0.07	0.68	.49	.03	[− .06, .12]	0.00	0.01	− 0.21	.84	− .01	[− .10, .08]
Female gender	0.69	0.55	1.24	.21	.10	[− .06, .26]	0.08	0.05	1.47	.14	.12	[− .04, .29]
**Non-binary gender**	**4.52**	**1.88**	**2.41**	**.02**	**.66**	**[ .12, 1.19]**	0.15	0.18	0.82	.41	.24	[− .33, .80]
Non-White minority	− 0.92	0.61	− 1.52	.13	− .13	[− .31,.04]	0.03	0.06	0.60	.55	.06	[− .13, .24]
**Parent (yes)**	** − 1.85**	**0.80**	**− 2.33**	**.02**	** − .27**	**[− .50, − .04]**	** − 0.22**	**0.08**	** − 2.91**	**.00**	** − .35**	**[− .59, − .11]**
Partnered (yes)	− 0.17	0.56	− 0.29	.77	− .02	[− .19, .14]	0.04	0.05	0.66	.51	.06	[− .11, .23]
**Educational attain**	** − 1.02**	**0.22**	** − 4.60**	**.00**	** − .20**	**[− .28, − .11]**	− 0.03	0.02	− 1.59	.11	− .07	[− .16, .02]
Childhood Income	0.34	0.37	0.94	.35	.04	[− .04, .12]	0.07	0.04	1.90	.06	.08	[.00, .17]

Note. Significant predictors and covariates are in bold

**Table 6:** Linear regression of anxiety symptoms and aggressive behavior on Centeredness and other variables in Sample 1

	Anxiety symptoms						Aggressive behavior					
Variable	*ß*	SE	*t*-value	*p* value	*F*^2^	*F*^2^ 95% CI	*ß*	SE	*t*-value	*p* value	*F*^2^	*F*^2^ 95% CI
(Intercept)	7.40	0.49	15.21	.00	− .01	[− .17, .15]	2.68	0.06	47.16	.00	.18	[.01, .34]
**Centeredness**	** − 0.09**	**0.02**	** − 3.59**	**.00**	** − .26**	**[− .40, − .12]**	** − 0.01**	**0.00**	** − 3.81**	**.00**	** − .29**	**[− .44, − .14]**
**ACEs**	**0.39**	**0.16**	**2.44**	**.02**	**.14**	**[.03, .26]**	0.01	0.02	0.49	.63	.03	[− .09, .15]
**BCEs**	** − 0.42**	**0.14**	** − 2.95**	**.00**	** − .15**	**[− .25, − .05]**	0.01	0.02	0.48	.63	.03	[− .08, .13]
Avoidance	− 0.22	0.28	− 0.78	.43	− .05	[− .18, .08]	− 0.01	0.03	− 0.29	.77	− .02	[− .16, .12]
Anxiety	− 0.09	0.23	− 0.39	.70	− .02	[− .13, .09]	0.04	0.03	1.56	.12	.09	[− .02, .21]
Age	0.00	0.06	− 0.04	.97	.00	[− .09, .09]	0.01	0.01	1.54	.12	.07	[− .02, .17]
**Female gender**	**1.00**	**0.49**	**2.03**	**.04**	**.17**	**[.01, .33]**	** − 0.20**	**0.06**	** − 3.50**	**.00**	** − .30**	**[− .47, − .13]**
**Non-binary gender**	**3.82**	**1.69**	**2.27**	**.02**	**.64**	**[.09, 1.20]**	0.20	0.20	1.00	.32	.30	[− .28, .88]
**Non-White minority**	** − 1.75**	**0.54**	** − 3.24**	**.00**	** − .30**	**[− .47, − .12]**	0.04	0.06	0.69	.49	.07	[− .12, .25]
**Parent (yes)**	** − 1.87**	**0.72**	** − 2.61**	**.01**	** − .31**	**[− .55, − .08]**	− 0.01	0.08	− 0.13	.90	− .02	[− .26, .23]
Partnered (yes)	0.48	0.50	0.96	.34	.08	[− .09, .25]	− 0.05	0.06	− 0.77	.44	− .07	[− .24, .11]
**Educational attain**	** − 0.42**	**0.20**	** − 2.12**	**.03**	** − .09**	**[− .18, − .01]**	** − 0.11**	**0.02**	** − 4.82**	**.00**	** − .22**	**[− .32, − .13]**
Childhood income	− 0.15	0.32	− 0.47	.64	− .02	[− .11, .07]	0.03	0.04	0.88	.38	.04	[− .05, .13]

Note. Significant predictors and covariates are in bold

**Table 7:** Linear regression of life satisfaction and the dimensional composite on Centeredness and other variables in Sample 1

	Life satisfaction							Composite outcome					
Variable	*ß*	SE	*t*-value	*p* value	*F*^2^	*F*^2^ 95% CI		*ß*	SE	*t*-value	*p* value	*F*^2^	*F*^2^ 95% CI
(Intercept)	16.97	0.64	26.54	.00	− 0.28	[− .44, − .13]		2.36	1.39	1.70	.09	0.14	[− .01, .29]
**Centeredness**	**0.08**	**0.03**	**2.49**	**.01**	**0.17**	**[.04, .31]**		** − 0.30**	**0.07**	** − 4.26**	**.00**	** − 0.29**	**[− .42, − .15]**
ACEs	0.04	0.21	0.21	.83	0.01	[− .10, .12]		0.74	0.46	1.59	.11	0.09	[− .02, .20]
**BCEs**	**0.63**	**0.19**	**3.36**	**.00**	**0.16**	**[.07, .26]**		** − 1.90**	**0.41**	** − 4.61**	**.00**	** − 0.21**	**[− .31, − .12]**
**Avoidance**	** − 1.17**	**0.36**	** − 3.23**	**.00**	** − 0.20**	**[− .33, − .08]**		0.66	0.79	0.84	.40	0.05	[− .07, .17]
Anxiety	0.12	0.30	0.40	.69	0.02	[− .08, .13]		− 0.32	0.66	− 0.48	.63	− 0.02	[− .13, .08]
**Age**	** − 0.16**	**0.08**	** − 2.02**	**.04**	** − 0.09**	**[− .17, .00]**		0.17	0.17	1.00	.32	0.04	[− .04, .13]
Female gender	0.39	0.64	0.60	.55	0.05	[− .11, .20]		0.94	1.41	0.67	.51	0.05	[− .10, .20]
Non-binary gender	0.26	2.21	0.12	.91	0.03	[− .50, .56]		8.38	4.74	1.77	.08	0.46	[− .05, .97]
Non-White minority	0.45	0.71	0.64	.52	0.06	[− .12, .23]		− 2.65	1.56	− 1.70	.09	− 0.15	[− .31, .02]
**Parent (yes)**	**4.25**	**0.93**	**4.57**	**.00**	**0.52**	**[.30, .74]**		** − 8.18**	**2.04**	** − 4.00**	**.00**	** − 0.45**	**[− .67, − .23]**
**Partnered (yes)**	**2.35**	**0.66**	**3.56**	**.00**	**0.29**	**[.13, .45]**		− 2.10	1.44	− 1.46	.15	− 0.12	[− .27, .04]
**Educational attain**	**0.92**	**0.26**	**3.56**	**.00**	**0.15**	**[.07, .23]**		** − 2.41**	**0.57**	** − 4.23**	**.00**	** − 0.18**	**[− .26, − .09]**
Childhood income	0.74	0.43	1.73	.08	0.07	[− .01, .15]		− 0.26	0.94	− 0.28	.78	− 0.01	[− .09, .07]

Note. Significant predictors and covariates are in bold

## Discussion

This study introduced the concept of Centeredness and examined the psychometric properties of a novel Centeredness scale compared to another well-established instrument assessing childhood relationships [(i.e., attachment-related avoidance and anxiety from the ECR-RS (Fraley et al., 2011)]. We also examined the predictive validity of overall Centeredness compared to both attachment constructs, as well as ACEs and BCEs. This allowed a test of whether the emotional *quality* of the childhood home environment predicted mental health outcomes over and above the *quantity* of adverse and benevolent childhood experiences. Broadly, results supported both the construct validity and the predictive validity of Centeredness scale for a range of young adulthood mental health outcomes, even when accounting for the other childhood predictors as well as sociodemographic covariates.

## Descriptive Findings on Centeredness Across Sociodemographic Groups

### Race/Ethnicity

In both samples, mean levels of Centeredness were comparable in individuals identifying as White, Black, Latine, and Asian. These findings indicate that overall Centeredness did not seem to differ according individuals’ racial/ethnic identities. These findings suggest that, similar to the BCEs scale (Narayan et al., 2018), the Centeredness scale may be culturally appropriate to use across these four racial/ethnic groups, as racial/ethnic identity itself does not seem to relate to different individual perceptions of Centeredness. It is important to note, however, that the majority of participants in both samples of this study identified as White (approximately two-thirds of each sample) and that the percentages of the three largest minority groups in this study were relatively small compared to the overall sample sizes. Further, all participants were drawn from the USA, regardless of their race or ethnicity. Thus, the lack of mean differences in Centeredness across racial/ethnic identity groups could be attributed to shared cultural factors of being born and residing in the USA. The Centeredness scale also needs to be tested across individuals who identify as other racial/ethnic groups and who reside in different countries than the USA.

### Gender Identity

Although Centeredness did not differ by participants’ racial/ethnic identity, mean levels of Centeredness did differ by gender identity. In both samples, individuals identifying as male reported their overall levels of Centeredness to be significantly higher than individuals identifying as female or gender non-conforming. This finding was unexpected but may reflect previous research that males report some of their life experiences more favorably than females (Sroufe et al., 2005; Werner & Smith, 2001). When post hoc analyses were conducted to further explore gender differences, the associations between Centeredness and all outcomes remained significant for both males and females when examined separately. (Individuals identifying as gender-non-conforming were not included in these correlation analyses due to extremely low cell sizes in both samples.) This finding tentatively suggests that Centeredness is a valid indicator of psychological adjustment for both males and females.

For each Centeredness item, mean differences were also examined across all three gender identity groups. Results in Sample 1 indicated that mean levels of certain Centeredness items were reported to be higher for males versus females (but not for males versus gender non-conforming or for female versus gender non-conforming individuals). These findings tentatively point to specific aspects of Centeredness that may be either experienced disparately across males and females during childhood and/or perceived more negatively by female adults versus male adults when they are looking back on their childhoods. For instance, it is possible that some primary caregivers and other family members may indeed be more likely to dismiss and not productively handle female children’s negative emotions compared to male children’s negative emotions. Furthermore, some female children may experience their home environments as more tense and unpredictable, less perfect, and as if they were outsiders compared to males. Alternatively, it is also possible that female adults may recall their family members as being more likely to act in these ways than male adults do even if the qualities of the actual childhood home environments were comparable. While his study is not able to clarify whether one or several of these possibilities is accurate, these are promising areas of future research with the Centeredness scale. Additionally, future research with greater numbers of individuals identifying as gender non-conforming is greatly needed, as descriptive findings from Sample 2 indicated differences in additional Centeredness items across all three gender identity groups. (These findings are not discussed here because these differences may have been in part due to Sample 2’s much larger size, which may have increased the odds of detecting significant but very small effects).

## Construct Validity of Centeredness

Findings revealed that higher levels of Centeredness were indeed significantly associated with lower levels of attachment-related avoidance and anxiety. Higher levels of Centeredness were also significantly related to lower levels of total ACEs and higher levels of total BCEs. These associations were all in the modest to substantial range in both samples (absolute values of all correlations of Centeredness and attachment problems, BCEs, and ACEs were between .56 and .75). These findings and the magnitude of these associations suggest that Centeredness assesses somewhat overlapping aspects of childhood experience and relationships compared to attachment-related avoidance, attachment-related anxiety, and the quantity of reported childhood adversities and positive childhood experiences. These findings also suggest that Centeredness assesses unique aspects that these other measures of childhood relationships and experiences do not assess. The emotional quality of the home environment, characterized by connectedness, support, belonging, and acceptance, is therefore a distinct and meaningful construct compared to childhood attachment relationships and the quantity of ACEs or BCEs.

## Predictive Validity of Centeredness

Higher levels of Centeredness significantly predicted each of the mental health outcomes in both samples. Specifically, higher levels of Centeredness predicted lower levels of depression and anxiety symptoms, STBs, and aggressive behavior, higher levels of life satisfaction, and lower levels of the dimensional composite. Centeredness was the only independent variable that predicted each of these outcomes across both samples when also accounting for all other childhood predictors and covariates. Importantly, however, when examining the magnitude of the predictive effects of Centeredness compared to other predictor (i.e., attachment-related problems, ACEs, and BCEs, and their *F*^2^ values in the regression tables) findings revealed the strength of the effects across predictors was comparable. In other words, Centeredness predicted more mental health outcomes (i.e., all six outcomes in both samples for a total of 12 possible outcomes) compared to ACEs (only three outcomes in Sample 1 and two of these in Sample 2) and to attachment-related avoidance and anxiety (neither of which consistently predicted any mental health outcome across both samples). However, when Centeredness and ACEs did predict the same outcome (i.e., depression and anxiety symptoms, and STBs) in Sample 1, the strength of all effects was generally comparable according to the magnitude of their effect sizes. This observation was also true for Centeredness versus BCEs; most significant effects for both predictors were medium in effect size. While Centeredness may predict more mental health outcomes than other predictors, it does not necessarily predict these outcomes more strongly.

Taken together, the finding that both Centeredness and BCEs predicted the greatest range of outcomes points to the importance of capturing both the emotional quality of positive home environments and family relationships (i.e., Centeredness) and the quantity of positive childhood experiences and resources (including within the family as well as with friends, teachers, and neighbors; i.e., BCEs or PCEs). Unexpectedly, neither attachment-related anxiety nor avoidance consistently predicted mental health outcomes when also accounting for Centeredness and BCEs. This may be because while attachment-related anxiety and avoidance are well-established constructs (Fraley et al., 2011), both reflect childhood relationship dysfunction, rather than positive aspects of relationships. It may also be the case that attachment-related problems were not predictive because this study measured them in regard to participants’ mothers/mother figures and fathers/father figures specifically (as opposed to modifying wording to retrospectively assess childhood attachment representations more globally across the household). The Centeredness scale, on the other hand, allowed participants to reflect on relationships with non-traditional primary caregivers (e.g., a grandmother) and across other family members living in the household (e.g., siblings, relatives). It is possible that this difference contributed to broader effects of Centeredness than attachment-related problems on mental health outcomes.

Childhood adversity also did not predict mental health outcomes as broadly as Centeredness (or BCEs). In both samples, higher levels of ACEs only predicted higher levels of depression and anxiety symptoms but not other outcomes. Notably, this lack of broader effects of ACEs was not because levels of ACEs were low. Roughly two-thirds of participants in Sample 1 and Sample 2 reported at least one ACE (71.2% and 62.2%, respectively) and roughly 20 to 25% of participants in both samples reported four or more ACEs (25.5% and 19.9%, respectively). Even though ACEs were prevalent, they were not as broadly predictive as Centeredness or BCEs. These findings somewhat contradict previous research linking ACEs to broad mental health problems (Dube et al., 2001, 2003; Felitti et al., 1998). However, very little research has examined links between ACEs and mental health outcomes while also accounting for positive childhood experiences (Bethell, 2019; Narayan et al., 2018). Future efforts to model the effects of childhood experiences across adulthood outcomes need to account for positive childhood experiences.

Finally, it is important to note that all findings regarding Centeredness held while accounting for several demographic covariates. None of these covariates were as broadly predictive of outcomes as Centeredness (or BCEs) in both samples. These findings further support Centeredness as a salient predictor of young adults’ mental health after accounting for both childhood and contemporaneous covariates.

## Implications of Centeredness for Young Adulthood Mental Health

Findings that these childhood assets are more broadly and consistently predictive of adulthood mental health outcomes than childhood adversities have several implications for family resilience. The majority of current mental health efforts to prevent ACEs and deter intergenerational trauma focus on reducing childhood adversity (CDC, 2021). However, these efforts should also focus on increasing positive childhood experiences (Narayan et al., 2021). In many samples, the association between childhood adversity and positive experiences is significant but modest (Morris et al., 2021; Narayan et al., 2018). Here, correlations (*r*) between total ACEs with Centeredness or total BCEs were all between the absolute values of .47 and .66, indicating that childhood adversity shared less than half of its variance (*r*^2^) with Centeredness and BCEs. Childhood adversity and positive childhood experiences are not mutually exclusive; in reality, many individuals experience high levels of both (Narayan et al., 2018, 2021). Furthermore, many studies have found that links between positive childhood experiences and better mental health are independent of links between childhood adversity and poorer mental health (Crandall et al., 2021; Narayan et al., 2018). Efforts to increase positive childhood experiences, including both the emotional quality of family relationships and the quantity of childhood resources, may be equally, if not more, important for long-term wellbeing than reducing the effects of adversity.

This message may be particularly hopeful for parents of children who have experienced adversity. Rather than believing that they must only fix or correct the effects of adversity, parents could also focus on strengthening what is already there, the bonds with their children. Psychoeducation and prevention efforts geared towards strengthening Centeredness could focus on helping parents to consistently support children’s distress; validate children’s full range of emotions; not react with dismissiveness, hostility, or abuse; value and delight in children’s pursuits and accomplishments; and unconditionally accept children’s identities and core beliefs. This focus on “strengthening the positive” rather than or in addition to “reducing or eliminating the negative” may represent a more accessible goal for many current parents.

## Strengths and Limitations

This study possesses several methodological strengths. Individuals completed the Centeredness scale by considering all the people whom they uniquely considered to be a part of their childhood household. These nuances allowed for respondents who had multiple home environments and/or caregivers to choose those whom they deemed most influential for their upbringing. This instrument allows for the composition of individuals’ childhood households and caregiving systems to flexibly vary across respondents without needing to administer several different versions of the same scale for each target relationship.

In terms of additional methodological strengths, we examined all hypotheses in both a test and replication sample, and all associations involving Centeredness and outcomes replicated across both samples. We also allowed for individuals to identify as gender non-conforming, a historically overlooked practice in longitudinal samples assessing childhood experiences and contemporaneous mental health problems (Sroufe et al., 2005; Werner & Smith, 2001). Finally, we controlled for several childhood and contemporaneous covariates that could influence mental health outcomes, and all findings held.

Despite these strengths, numerous limitations existed. First and foremost, all childhood experiences were assessed, retrospectively. It is possible that contemporaneous influences that were unaccounted for (e.g., current stress and personality factors) influenced the strength of associations between Centeredness and other childhood predictors with mental health outcomes. However, the magnitude of the associations between Centeredness and the internalizing problems that are often associated with retrospective reporting biases (e.g., depression and anxiety; Reuben et al., 2016), were not substantially different in size than the associations between Centeredness and the non-internalizing outcomes (e.g., aggression and life satisfaction). This finding suggests that participants’ internalizing distress was unlikely to have strongly affected their retrospective reports of Centeredness. We were also unable to assess the test–retest reliability of the Centeredness scale due to constraints of the overall study design, which should be a priority for future research. Despite the lack of test–retest reliability, evidence for replicability of the effects of Centeredness across two independent samples was promising.

In terms of sampling limitations, neither sample was representative of the young adults in the USA, although samples drew from individuals living in all 50 states. Additionally, the majority of participants in both samples identified as White and as either male or female. Both samples lacked adequate representation of individuals who identified as being from racial/ethnic minority groups, and a major limitation was that the number of people identifying as gender minorities was particularly small. Samples that have higher proportions of several different types of minority groups, including individuals from other countries, would be better equipped to examine whether overall Centeredness is culturally generalizable and shows measurement invariance across individuals who identify as being part of one or more minority group. For instance, with greater representation of minoritized individuals, future studies could examine if associations between overall Centeredness and each mental health problem were similar in magnitude depending on different cultural dimensions.

In terms of other future directions, other studies in diverse samples should continue to examine the factor structure of the Centeredness scale. The present study did not set out to detect subfactors of Centeredness (and current evidence for multiple factors was not clear nor convincing), so this is a viable area for future research endeavors. Finally, this study did not explicitly test whether Centeredness is easily translatable to be used by clinical or community providers who work directly with parents to strengthen relationships and emotional bonds with children. Especially given the translational nature of the questions and the goals of this measure, a next step in this direction would be to have a large sample of parents from diverse backgrounds complete the Centeredness scale and provide feedback on whether it elicits their insight about how to recreate positive relationships with current children and/or create new positive experiences.

## Conclusions

The effects of positive childhood experiences and relationships, including the quality and quantity of them, on young adults’ mental health may be equally if not more important to assess than the effects of childhood adversity and attachment-related problems. Efforts to prevent long-term consequences of childhood toxic stress on adulthood maladjustment may be missing half the story if they neglect to account for the role of positive childhood experiences and relationships. This study illuminates a hopeful message to adults and parents, as well as researchers and policy makers, who wish to provide their own children with positive experiences and relationships to shape long-term healthy development. Strengthening emotional bonds and connectedness between parents and children, fostering children’s perceived belongingness, and promoting unconditional parental acceptance of children’s identities and core beliefs may be antidotes to childhood adversity that can have lasting benefits on more resilient functioning into adulthood.

## Supplementary Information

**Additional file 1: Table 8.** Centeredness Items and Gender Differences for Sample 2. **Table 9.** Bivariate Correlations between Childhood Predictors and Mental Health Outcomes in Sample 2. **Table 10.** Bivariate Correlations between Childhood Predictors and Demographic Covariates in Sample 1.** Table 11.** Bivariate Correlations between Childhood Predictors and Demographic Covariates in Sample 2. **Table 12.** Linear Regression of Depression Symptoms and STBs on Centeredness and Other Variables in Sample 2. **Table 13.** Linear Regression of Anxiety Symptoms and Aggressive Behavior on Centeredness and Other Variables in Sample 2. **Table 14.** Linear Regression of Life Satisfaction and the Dimensional Composite on Centeredness and Other Variables in Sample 2. **Figure 2.** Confirmatory Factor Analysis and Factor Structure for the Final 20 Centeredness Items. (DOCX 142 kb)

## Data Availability

Data and analysis code for this study are available upon request by emailing the corresponding author.
