# Supplementary material for: Childhood Centeredness is a Broader Predictor of Young Adulthood Mental Health than Childhood Adversity, Attachment, and Other Positive Childhood Experiences
Source: Advers Resil Sci. 2023 Mar 23;4(2):191–210. doi: 10.1007/s42844-023-00089-x (PMC10033291; doi:10.1007/s42844-023-00089-x)

**Supplemental Material**

The supplemental material includes: Descriptive statistics for gender differences between all three gender identity groups across all Centeredness items (Table 8), bivariate correlations between childhood predictors and young adulthood mental health outcomes from Sample 2 (Table 9), bivariate correlations between childhood predictors and all sociodemographic covariates from Sample 1 (Table 10), and Sample 2 (Table 11), regression analyses for all mental health outcomes from Sample 2 (Tables 12-14), and results depicting the factor structure of the 20 Centeredness items from the CFA in Sample 2 (Figure 2).

**Table 8.** *Centeredness Items and Gender Differences for Sample 2*

| **Item *#*** | **Item Wording** | Male (n = 497) | Female (n = 673) | Gender Non-Confirming (n = 28) | ANOVA *p*-value | Sign. contrast (*p* < .017) |
| --- | --- | --- | --- | --- | --- | --- |
| 1 | After a bad day, I could count on my family to make me feel better. | 3.5 (1.2) | 3.3 (1.2) | 2.8 (1.0) | <.001 | M vs. NC |
| 2 | I felt like an outsider in my family. (reversed) | 3.7 (1.3) | 3.3 (1.3) | 2.6 (1.2) | <.001 | M vs. F; M vs. NC; F vs. NC |
| 3 | My home and family were perfect. | 3.0 (1.2) | 2.5 (1.2) | 2.1 (1.1) | <.001 | M vs. F; M vs. NC |
| 4 | When I came home at the end of a long day, I expected my home environment to feel tense or unpredictable. (reversed) | 3.5 (1.2) | 3.2 (1.3) | 2.7 (1.4) | <.001 | M vs. F; M vs. NC |
| 5 | I felt like my emotions were dismissed as incorrect (e.g., "You are overreacting"). (reversed) | 3.3 (1.2) | 2.7 (1.3) | 2.1 (1.1) | <.001 | M vs. F; M vs. NC |
| 6 | My family valued my input. | 3.4 (1.2) | 3.1 (1.1) | 2.6 (.8) | <.001 | M vs. F; M vs. NC |
| 7 | I felt unnoticed when I was around my family. (reversed) | 3.9 (1.1) | 3.6 (1.2) | 3.4 (1.2) | <.001 | M vs. F |
| 8 | I felt completely satisfied with my home and my family. | 3.4 (1.2) | 3.0 (1.3) | 2.4 (1.2) | <.001 | M vs. F; M vs. NC |
| 9 | I was nervous that someone in my family would say or do something hurtful. (reversed) | 3.6 (1.3) | 3.1 (1.3) | 2.6 (1.4) | <.001 | M vs. F; M vs. NC |
| 10 | I received enough one-on-one time with my parents. | 3.8 (1.1) | 3.5 (1.2) | 3.1 (1.1) | <.001 | M vs. F; M vs. NC |
| 11 | My parents were frequently and easily upset. (reversed) | 3.3 (1.2) | 2.8 (1.3) | 2.5 (1.3) | <.001 | M vs. F; M vs. NC |
| 12 | When I was upset, I felt like my parents tried to find a way to be on my side. | 3.4 (1.1) | 3.1 (1.2) | 2.6 (1.2) | <.001 | M vs. F; M vs. NC |
| 13 | My parents believed that I made good choices. | 3.7 (1.1) | 3.6 (1.1) | 3.6 (1.1) | .53 | -- |
| 14 | My parents pointed out positive things about me. | 3.8 (1.1) | 3.6 (1.2) | 3.5 (1.0) | <.001 | M vs. F |
| 15 | My parents tried to understand how I was feeling by putting themselves in my shoes. | 3.1 (1.1) | 2.6 (1.2) | 2.1 (1.1) | <.001 | M vs. F; M vs. NC |
| 16 | It would hurt our relationship if I chose different views/beliefs (e.g., religious, political, etc.) than my parents. (reversed) | 3.1 (1.3) | 2.8 (1.3) | 2.5 (1.4) | <.001 | M vs. F |
| 17 | I felt like my parents were happy to see me when I came home after being gone for the day. | 3.9 (1.0) | 3.7 (1.1) | 3.5 (1.0) | <.001 | M vs. F |
| 18 | When I was upset, I felt like my parents couldn’t handle my negative emotions (e.g., they left the room or told me I shouldn’t feel that way). (reversed) | 3.2 (1.2) | 2.8 (1.3) | 2.4 (1.1) | <.001 | M vs. F; M vs. NC |
| 19 | My parents mentioned ways they were proud of me to other people (e.g., family members, their friends, other adults, etc.). | 4.0 (1.1) | 3.8 (1.1) | 4.0 (.8) | .03 | -- |
| 20 | My parents had views (religious, political, racial, cultural, etc.) that made me feel hesitant to express in front of others because I didn't agree with them, or I worried that others would not agree with them. (reversed) | 3.3 (1.3) | 3.2 (1.3) | 2.3 (1.5) | <.001 | M vs. NC; F vs. NC |

*Note***.** Sign. contrast shows pairwise contrasts significant at *p* < .017 across the three gender groups (M = Male, F = Female, NC = Gender Non-Conforming).

**Table 9.** *Bivariate Correlations between Childhood Predictors and Mental Health Outcomes in Sample 2*

| **Independent Variables (Childhood Predictors)** | 1 | 2 | 3 | 4 | 5 | 6 | 7 | 8 | 9 | 10 | 11 |
| --- | --- | --- | --- | --- | --- | --- | --- | --- | --- | --- | --- |
| 1. Centeredness | - | .00 | .00 | .00 | .00 | .00 | .00 | .00 | .00 | .00 | .00 |
| 2. ACEs | -.66 | - | .00 | .00 | .00 | .00 | .00 | .00 | .00 | .00 | .00 |
| 3. BCEs | .61 | -.55 | - | .00 | .00 | .00 | .00 | .00 | .00 | .00 | .00 |
| 4. Avoidance | -.78 | .58 | -.57 | - | .00 | .00 | .00 | .00 | .00 | .00 | .00 |
| 5. Anxiety | -.67 | .63 | -.50 | .65 | - | .00 | .00 | .00 | .00 | .00 | .00 |
| **Dependent Variables**  **(Mental Health Outcomes)** |  |  |  |  |  |  |  |  |  |  |  |
| 6. Depression symptoms | -.33 | .29 | -.34 | .28 | .32 | - | .00 | .00 | .00 | .00 | .00 |
| 7. STBs | -.42 | .37 | -.38 | .33 | .37 | .59 | - | .00 | .00 | .00 | .00 |
| 8. Anxiety symptoms | -.45 | .41 | -0.38 | .36 | .38 | .78 | .45 | - | .00 | .00 | .00 |
| 9. Aggressive behavior | -.29 | .27 | -0.29 | .22 | .30 | .34 | .31 | .27 | - | .00 | .00 |
| 10. Life satisfaction | .44 | -.33 | 0.42 | -.39 | -.30 | -.54 | -.41 | -.46 | -.33 | - | .00 |
| 11. Overall Composite | -.53 | .45 | -0.49 | .46 | .42 | .90 | .59 | .84 | .41 | -.81 | - |

*Note*. Exact correlation coefficients are shown below the diagonal and exact p-values are shown above the diagonal.

**Table 10.** *Bivariate Correlations between Childhood Predictors and Demographic Covariates in Sample 1*

| **Independent Variables (Childhood Predictors)** | 1 | 2 | 3 | 4 | 5 | 6 | 7 | 8 | 9 | 10 | 11 | 12 | 13 | 14 |
| --- | --- | --- | --- | --- | --- | --- | --- | --- | --- | --- | --- | --- | --- | --- |
| 1. Centeredness | - | .00 | .00 | .00 | .00 | .85 | .01 | .00 | .48 | .61 | .70 | .10 | .02 | .00 |
| 2. ACEs | -.66 | - | .00 | .00 | .00 | .45 | .04 | .04 | .97 | .60 | .01 | .07 | .00 | .00 |
| 3. BCEs | .56 | -.47 | - | .00 | .00 | .25 | .84 | .95 | .61 | .76 | .15 | .06 | .00 | .00 |
| 4. Avoidance | -.75 | .53 | -.49 | - | .00 | .10 | .00 | .01 | 1.00 | .02 | .52 | .85 | .04 | .00 |
| 5. Anxiety | -.62 | .61 | -.42 | .61 | - | .99 | .04 | .05 | .92 | .98 | .12 | .11 | .02 | .00 |
| **Demographic Covariates** |  |  |  |  |  |  |  |  |  |  |  |  |  |  |
| 6. Age | -.01 | .03 | -.05 | .00 | -.07 | - | .75 | 1.00 | .28 | .00 | .00 | .00 | .00 | .21 |
| 7. Male Gender | .12 | -.09 | -.01 | -.12 | -.09 | .01 | - | .00 | .00 | .38 | .01 | .01 | .07 | .20 |
| 8. Female Gender | -.12 | .09 | .00 | .12 | .09 | .00 | -.96 | - | .00 | .33 | .01 | .02 | .06 | .17 |
| 9. Non-Binary Gender | .03 | .00 | .02 | .00 | .00 | -.05 | -.13 | .16 | - | .72 | .49 | .79 | .73 | .75 |
| 10. Non-White Minority | .02 | -.02 | .01 | .10 | .00 | -.19 | .04 | -.04 | .02 | - | .01 | .00 | .15 | .01 |
| 11. Parent (Yes) | -.02 | .12 | .06 | -.03 | .07 | .29 | -.11 | .12 | -.03 | -.12 | - | .00 | .45 | .03 |
| 12. Partnered (Yes) | -.07 | .08 | .08 | .01 | .07 | .15 | -.11 | .10 | .01 | -.21 | .24 | - | .00 | .93 |
| 13. Educational Attainment | .10 | -.16 | .18 | -.09 | -.10 | .33 | -.08 | .08 | -.01 | -.06 | .03 | .12 | - | .00 |
| 14. Childhood Income | .24 | -.36 | .24 | -.21 | -.25 | -.05 | -.05 | .06 | -.01 | -.12 | -.09 | .00 | .21 | - |

*Note*. Exact correlation coefficients are shown below the diagonal and exact p-values are shown above the diagonal.

**Table 11.** *Bivariate Correlations between Childhood Predictors and Demographic Covariates in Sample 2*

| **Independent Variables (Childhood Predictors)** | | 1 | 2 | 3 | 4 | 5 | 6 | 7 | 8 | 9 | 10 | 11 | 12 | 13 | 14 |  |
| --- | --- | --- | --- | --- | --- | --- | --- | --- | --- | --- | --- | --- | --- | --- | --- | --- |
| 1. Centeredness | - | | .00 | .00 | .00 | .00 | .25 | .00 | .00 | .00 | .02 | .13 | .03 | .00 | .00 |  |
| 2. ACEs | -.66 | | - | .00 | .00 | .00 | .33 | .00 | .00 | .01 | .52 | .15 | .61 | .00 | .00 |  |
| 3. BCEs | .61 | | -.55 | - | .00 | .00 | .67 | .03 | .17 | .01 | .93 | .14 | .01 | .00 | .00 |  |
| 4. Avoidance | -.78 | | .58 | -.57 | - | .00 | .11 | .00 | .00 | .00 | .02 | .02 | .00 | .00 | .00 |  |
| 5. Anxiety | -.67 | | .63 | -.50 | .65 | - | .37 | .00 | .00 | .18 | .77 | .03 | .22 | .00 | .00 |  |
| **Demographic Covariates** |  | |  |  |  |  |  |  |  |  |  |  |  |  |  |  |
| 6. Age | .03 | | .03 | .01 | -.05 | .03 | - | .62 | .94 | .06 | .00 | .00 | .00 | .00 | .01 |  |
| 7. Male Gender | .19 | | -.13 | .07 | -.17 | -.12 | .01 | - | .00 | .00 | .11 | .03 | .00 | .07 | .81 |  |
| 8. Female Gender | -.16 | | .11 | -.04 | .14 | .10 | .00 | -.95 | - | .00 | .13 | .00 | .00 | .05 | .85 |  |
| 9. Non-Binary Gender | -.09 | | .08 | -.08 | .09 | .04 | -.05 | -.13 | -.18 | - | .88 | .03 | .34 | .55 | .88 |  |
| 10. Non-White Minority | -.07 | | -.02 | .00 | .07 | .01 | -.23 | .05 | -.04 | .00 | - | .00 | .00 | .01 | .00 |  |
| 11. Parent (Yes) | .04 | | .04 | .04 | -.07 | .06 | .37 | -.06 | .08 | -.06 | -.11 | - | .00 | .01 | .01 |  |
| 12. Partnered (Yes) | .07 | | -.02 | .08 | -.12 | -.04 | .26 | -.10 | .10 | .03 | -.17 | .31 | - | .00 | .06 |  |
| 13. Educational Attainment | .19 | | -.25 | .29 | -.20 | -.15 | .39 | -.05 | .06 | -.02 | -.08 | .08 | .25 | - | .00 |  |
| 14. Childhood Income | .29 | | -.43 | .30 | -.29 | -.25 | -.07 | .01 | -.01 | .00 | -.10 | -.07 | .05 | .19 | - |  |

*Note*. Exact correlation coefficients are shown below the diagonal and exact p-values are shown above the diagonal.

**Table 12**. *Linear Regression of Depression Symptoms and STBs on Centeredness and Other Variables in Sample 2*

|  | Depression Symptoms | | | | | | Suicidal Thoughts and Behaviors (STBs) | | | | | |
| --- | --- | --- | --- | --- | --- | --- | --- | --- | --- | --- | --- | --- |
| Variable | *ß* | SE | t-value | p-value | F^2^ | F^2^ 95% CI | *ß* | SE | t-value | p-value | F^2^ | F^2^ 95% CI |
| (Intercept) | 9.57 | 0.38 | 25.41 | .00 | .10 | [-.01, .21] | 0.45 | 0.04 | 12.36 | .00 | .10 | [-.01, .22] |
| **Centeredness** | **-0.08** | **0.02** | **-4.23** | **.00** | **-.20** | **[-.30, -.11]** | **-0.01** | **0.00** | **-4.55** | **.00** | **-.23** | **[-.32, -.13]** |
| **ACEs** | **0.36** | **0.12** | **2.95** | **.00** | **.12** | **[ .04, .19]** | 0.01 | 0.01 | 1.03 | .30 | .04 | [-.04, .12] |
| **BCEs** | **-0.58** | **0.11** | **-5.23** | **.00** | **-.18** | **[-.25, -.11]** | **-0.04** | **0.01** | **-3.29** | **.00** | **-.12** | **[-.19, -.05]** |
| **Avoidance** | -0.18 | 0.21 | -0.85 | .40 | -.04 | [-.12, .05] | **-0.04** | **0.02** | **-1.83** | **.07** | **-.08** | **[-.18, .01]** |
| **Anxiety** | **0.49** | **0.18** | **2.65** | **.01** | **.10** | **[ .03, .18]** | **0.07** | **0.02** | **3.77** | **.00** | **.15** | **[.07, .23]** |
| **Age** | **-0.09** | **0.05** | **-1.99** | **.05** | **-.06** | **[-.13, .00]** | 0.00 | 0.00 | -0.09 | .93 | .00 | [-.07, .06] |
| **Female Gender** | **1.19** | **0.37** | **3.18** | **.00** | **.18** | **[.07, .28]** | 0.05 | 0.04 | 1.29 | .20 | .07 | [-.04, .19] |
| Non-Binary Gender | 1.47 | 1.16 | 1.26 | .21 | .22 | [-.12, .55] | 0.07 | 0.11 | 0.59 | .55 | .11 | [-.25, .47] |
| **Non-White Minority** | **-1.30** | **0.39** | **-3.34** | **.00** | **-.19** | **[-.31, -.08]** | **-0.09** | **0.04** | **-2.39** | **.02** | **-.14** | **[-.26, -.03]** |
| Parent (Yes) | -0.36 | 0.51 | -0.71 | .48 | -.05 | [-.20, .09] | 0.04 | 0.05 | 0.80 | .43 | .06 | [-.09, .22] |
| **Partnered (Yes)** | **-1.47** | **0.39** | **-3.73** | **.00** | **-.22** | **[-.33, -.10]** | **-0.12** | **0.04** | **-3.11** | **.00** | **-.19** | **[-.31, -.07]** |
| **Educational Attain.** | **-0.34** | **0.16** | **-2.15** | **.03** | **-.07** | **[-.13, -.01]** | **-0.07** | **0.02** | **-4.30** | **.00** | **-.14** | **[-.20, -.08]** |
| Childhood Income | -0.24 | 0.25 | -0.94 | .35 | -.03 | [-.09, .03] | -0.04 | 0.02 | -1.79 | .07 | -.06 | [-.12, .01] |

*Note*. Significant predictors and covariates are in bolded black.

**Table 13**. *Linear Regression of Anxiety Symptoms and Aggressive Behavior on Centeredness and Other Variables in Sample 2*

|  | Anxiety Symptoms | | | | | | Aggressive Behavior | | | | | |
| --- | --- | --- | --- | --- | --- | --- | --- | --- | --- | --- | --- | --- |
| Variable | *ß* | SE | t-value | p-value | F^2^ | F^2^ 95% CI | *ß* | SE | t-value | p-value | F^2^ | F^2^ 95% CI |
| (Intercept) | 7.20 | 0.34 | 21.39 | .00 | -.02 | [-.14, .09] | 2.61 | 0.04 | 59.93 | .00 | .11 | [-.01, .23] |
| **Centeredness** | **-0.08** | **0.02** | **-5.15** | **.00** | **-.25** | **[-.35, -.16]** | **-0.01** | **0.00** | **-3.89** | **.00** | **-.20** | **[-.31, -.10]** |
| **ACEs** | **0.33** | **0.11** | **3.00** | **.00** | **.12** | **[.04, .20]** | 0.01 | 0.01 | 0.53 | .59 | .02 | [-.06, .11] |
| **BCEs** | **-0.34** | **0.10** | **-3.45** | **.00** | **-.12** | **[-.20, -.05]** | **-0.04** | **0.01** | **-3.18** | **.00** | **-.12** | **[-.20, -.05]** |
| **Avoidance** | -0.26 | 0.18 | -1.42 | .16 | -.06 | [-.15, .02] | **-0.06** | **0.02** | **-2.37** | **.02** | **-.11** | **[-.21, -.02]** |
| **Anxiety** | **0.42** | **0.16** | **2.54** | **.01** | **.10** | **[.02, .18]** | **0.08** | **0.02** | **3.83** | **.00** | **.16** | **[ .08, .25]** |
| **Age** | **-0.10** | **0.04** | **-2.40** | **.02** | **-.08** | **[-.14, -.01]** | 0.00 | 0.01 | -0.92 | .36 | -.03 | [-.10, .04] |
| **Female Gender** | **1.53** | **0.33** | **4.62** | **.00** | **.26** | **[.15, .37]** | **-0.22** | **0.04** | **-5.01** | **.00** | **-.30** | **[-.42, -.18]** |
| Non-Binary Gender | 0.93 | 1.04 | 0.89 | .37 | .16 | [-.19, .50] | -0.19 | 0.13 | -1.42 | .16 | -.27 | [-.64, .10] |
| **Non-White Minority** | **-1.44** | **0.35** | **-4.14** | **.00** | **-.25** | **[-.36, -.13]** | 0.05 | 0.05 | 1.20 | .23 | .08 | [-.05, .20] |
| **Parent (Yes)** | -0.53 | 0.45 | -1.17 | .24 | -.09 | [-.24, .06] | **0.20** | **0.06** | **3.35** | **.00** | **.27** | **[.11, .43]** |
| Partnered (Yes) | -0.29 | 0.35 | -0.82 | .41 | -.05 | [-.17, .07] | -0.01 | 0.05 | -0.29 | .78 | -.02 | [-.14, .11] |
| **Educational Attain.** | -0.06 | 0.14 | -0.46 | .65 | -.01 | [-.08, .05] | **-0.07** | **0.02** | **-3.80** | **.00** | **-.13** | **[-.19, -.06]** |
| Childhood Income | -0.11 | 0.22 | -0.48 | .63 | -.01 | [-.07, .04] | 0.03 | 0.03 | 0.95 | .34 | .03 | [-.03, .09] |

*Note.* Significant predictors and covariates are in bolded black.

**Table 14**. *Linear Regression of Life Satisfaction and the Dimensional Composite on Centeredness and Other Variables in Sample 2*

|  | Life Satisfaction | | | | | | Composite Outcome | | | | | |
| --- | --- | --- | --- | --- | --- | --- | --- | --- | --- | --- | --- | --- |
| Variable | *ß* | SE | t-value | p-value | F^2^ | F^2^ 95% CI | *ß* | SE | t-value | p-value | F^2^ | F^2^ 95% CI |
| (Intercept) | 17.96 | 0.42 | 42.55 | .00 | -.38 | [-.49, -.27] | 3.28 | 0.96 | 3.40 | .00 | .20 | [.09,.30] |
| **Centeredness** | **0.11** | **0.02** | **5.13** | **.00** | **.24** | **[ .15, .33]** | **-0.28** | **0.05** | **-5.99** | **.00** | **-.27** | **[-.36, -.18]** |
| **ACEs** | 0.00 | 0.14 | 0.03 | .98 | .00 | [-.07, .08] | **0.73** | **0.31** | **2.34** | **.02** | **.09** | **[.01, .16]** |
| **BCEs** | **0.61** | **0.12** | **4.93** | **.00** | **.17** | **[.10, .24]** | **-1.73** | **0.28** | **-6.11** | **.00** | **-.20** | **[-.27, -.14]** |
| Avoidance | -0.20 | 0.23 | -0.87 | .38 | -.04 | [-.12, .05] | -0.10 | 0.26 | -0.38 | .70 | -.02 | [-.10, .07] |
| Anxiety | -0.06 | 0.21 | -0.27 | .78 | -.01 | [-.08, .06] | 0.38 | 0.23 | 1.62 | .11 | .06 | [-.01, .13] |
| **Age** | **-0.19** | **0.05** | **-3.67** | **.00** | **-.11** | **[-.18, -.05]** | -0.01 | 0.12 | -0.10 | .92 | .00 | [-.06, .06] |
| **Female Gender** | 0.51 | 0.42 | 1.21 | .23 | .07 | [-.04, .17] | **2.03** | **0.95** | **2.13** | **.03** | **.11** | **[.01, .22]** |
| Non-Binary Gender | 0.29 | 1.30 | 0.22 | .82 | .04 | [-.29, .37] | 1.96 | 2.99 | 0.66 | .51 | .11 | [-.22, .43] |
| **Non-White Minority** | -0.47 | 0.44 | -1.09 | .28 | -.06 | [-.17, .05] | **-2.21** | **0.99** | **-2.22** | **.03** | **-.12** | **[-.23, -.01]** |
| **Parent (Yes)** | **2.05** | **0.57** | **3.62** | **.00** | **.26** | **[.12, .41]** | **-2.82** | **1.29** | **-2.19** | **.03** | **-.16** | **[-.30, -.02]** |
| **Partnered (Yes)** | **4.07** | **0.44** | **9.23** | **.00** | **.52** | **[.41, .64]** | **-5.75** | **1.00** | **-5.73** | **.00** | **-.32** | **[-.43, -.21]** |
| **Educational Attain.** | **0.75** | **0.18** | **4.29** | **.00** | **.13** | **[.07, .19]** | **-1.19** | **0.40** | **-2.96** | **.00** | **-.09** | **[-.14, -.03]** |
| Childhood Income | 0.54 | 0.28 | 1.90 | .06 | .05 | [.00, .11] | -0.95 | 0.64 | -1.49 | .14 | -.04 | [-.10, .01] |

*Note.* Significant predictors and covariates are in bolded black.

Figure 2. *Confirmatory Factor Analysis and Factor Structure for the Final 20 Centeredness Items.*


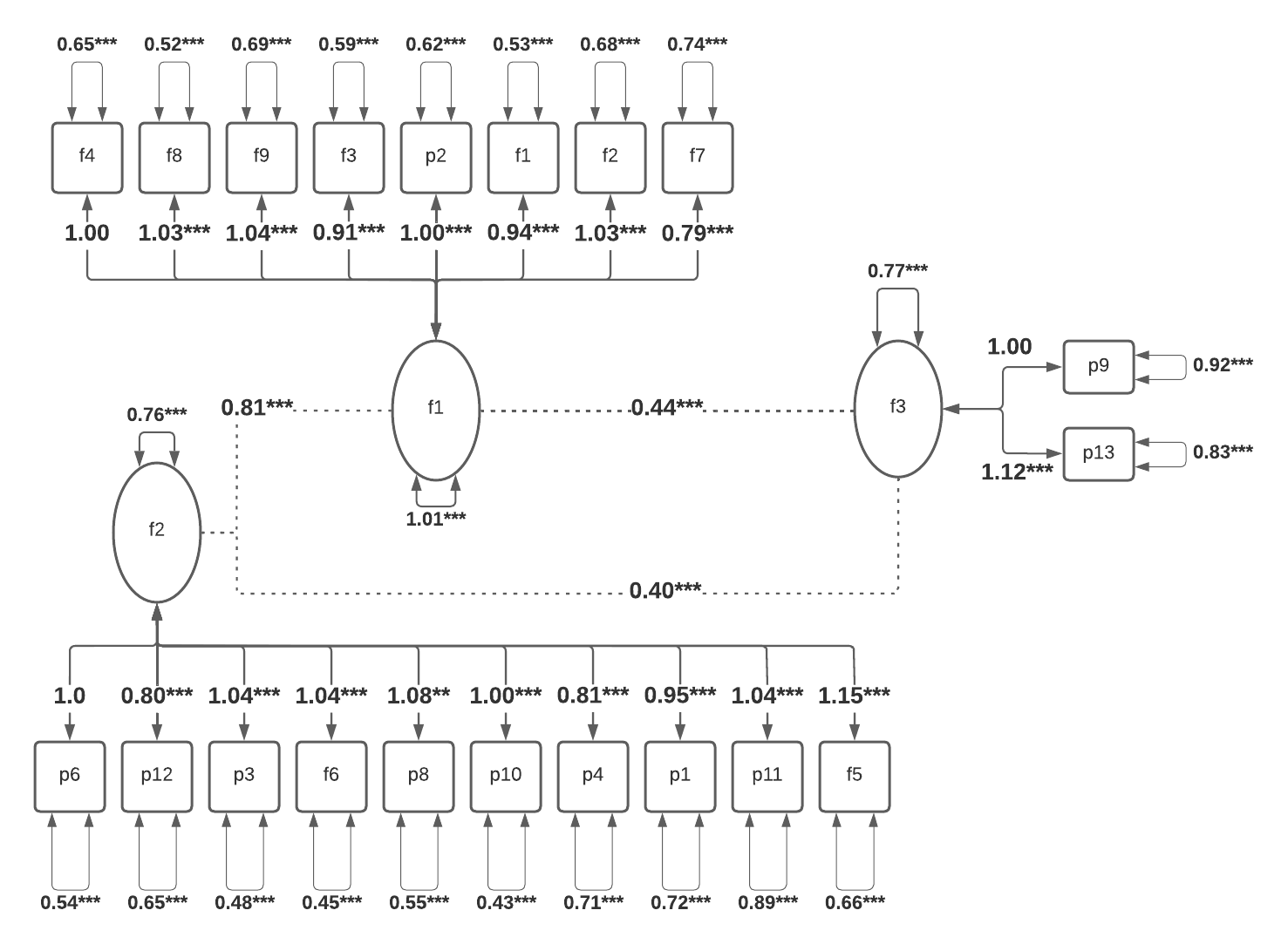

Supplement: Supplementary file 1 — Additional file 1: Table 8. Centeredness Items and Gender Differences for Sample 2. Table 9. Bivariate Correlations between Childhood Predictors and Mental Health Outcomes in Sample 2. Table 10. Bivariate Correlations between Childhood Predictors and Demographic Covariates in Sample 1. Table 11. Bivariate Correlations between Childhood Predictors and Demographic Covariates in Sample 2. Table 12. Linear Regression of Depression Symptoms and STBs on Centeredness and Other Variables in Sample 2. Table 13. Linear Regression of Anxiety Symptoms and Aggressive Behavior on Centeredness and Other Variables in Sample 2. Table 14. Linear Regression of Life Satisfaction and the Dimensional Composite on Centeredness and Other Variables in Sample 2. Figure 2. Confirmatory Factor Analysis and Factor Structure for the Final 20 Centeredness Items. (DOCX 142 kb) [file 42844_2023_89_MOESM1_ESM.docx]
